# Metabolomics profiling and neuroprotective effects of *Lagerstroemia loudonii* leaf extract and its kleptose Crysmeb^®^- stabilized loaded nanosuspension in seizure mice model

**DOI:** 10.1007/s11011-025-01756-x

**Published:** 2025-12-17

**Authors:** Sara M. Baraka, Nesma M.E. Abo El-Nasr, Rabab Kamel, Marwa M. Elbatanony, Omar A. Ahmed-Farid, Reda M. S. Korany, Salma A. El Sawi, Amal A. Maamoun

**Affiliations:** 1 Chemistry of Natural Compounds Department, National Research Centre, Giza, 12622 Egypt; 2 Pharmacology Department, Medical Research and Clinical Studies Institute, National Research Centre, Giza, 12622 Egypt; 3 Pharmaceutical Technology Department, National Research Centre, Giza, 12622 Egypt; 4 Pharmacognosy Department, National Research Centre, 33 Elbouhoth Street. Dokki, Cairo, 12622 Egypt; 5 Physiology Department, National Organization for Drug Control and Research, Giza, Egypt; 6 Pathology Department, Faculty of Veterinary Medicine, Cairo University, Giza, Egypt

**Keywords:** *Lagerstroemia loudonii*, UPLC-MS/MS, Epilepsy, Antioxidant, Neuroinflammation, Cyclodextrin, Nanosuspension

## Abstract

This research was designed to explore the anti-epileptic action of the ethanolic leaf extract of *Lagerstroemia loudonii* (LLE) compared to the extract-loaded MβCD-stabilized nanosuspension (MβCD-NS). UPLC–ESI–QTOF –MS/MS analysis of LLE revealed the identification of 57 compounds; phenolics were the major recorded class. Nanosuspensions were prepared and examined; the particle size of the selected physically stable MβCD-NS was 445.8 ± 61.08 nm with a uniform size distribution and an almost spherical outline as shown by TEM. Based on the acute toxicity results, LLE or MβCD-NS was orally gavage at doses of 100 and 200 mg/kg. Convulsions were induced by maximum electric shock (MES, 35 mA, 3 s), and pentylenetetrazole (PTZ, 85 mg/kg i.p) in mice. MβCD-NS (200 mg/kg) demonstrated significant anticonvulsant effects, including complete protection% in MES study and low tonic convulsion score in PTZ-model. PTZ injection instigated locomotor deficits, oxidative and nitrosative insults, neuroinflammation, imbalance in brain neurotransmitters viz. dopamine, serotonin, norepinephrine, GABA, glutamate, and severe structural changes in the brain. However, LLE and MβCD-NS (200 mg/kg) ameliorated all these alterations through preserving antioxidant defense mechanisms by improving GSH content, and Nrf2 immuno-expression, as well as reducing MDA, NO, and GSSG levels in the brain. MβCD-NS restored the brain neurotransmitter levels that involved in the observed improvement in the animals’ locomotor activity. The detected low TNF-α immuno-expression of the brain regions of the MβCD-NS group highlighted its anti-inflammatory action. In conclusion, MβCD-NS exhibited superior anti-epileptic potential by counteracting neuronal oxido-inflammatory damage, positioning it as a promising therapeutic strategy for epilepsy.

**Graphical Abstract:**

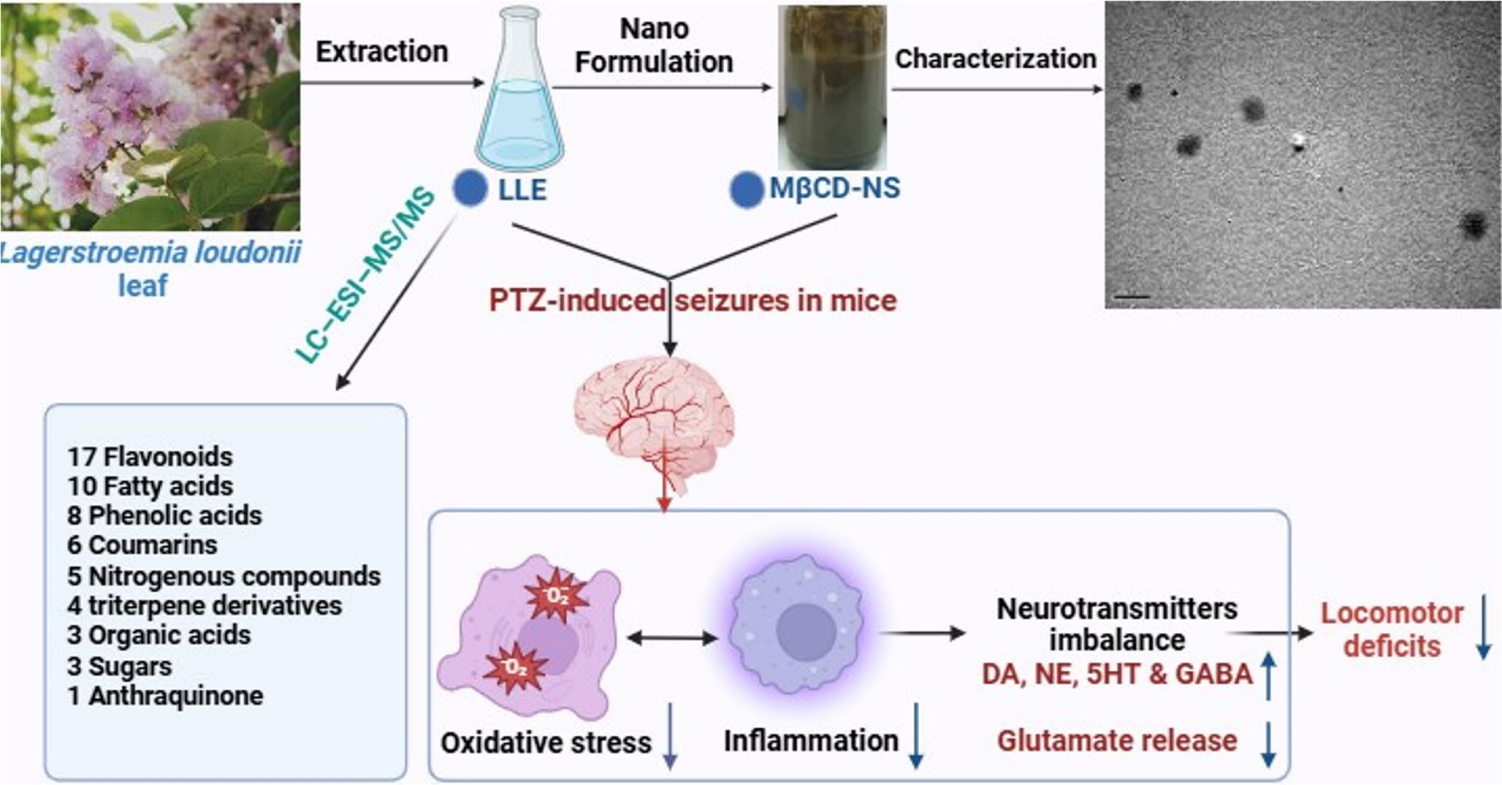

**Supplementary Information:**

The online version contains supplementary material available at 10.1007/s11011-025-01756-x.

## Introduction

Epilepsy is a fatal brain disorder resulting from excessive neuronal discharges that impair mobility, sensory, autonomic, and mental functioning in addition to causing uncontrollable seizures and attacks (Yuen et al. 2018). Over 50 million patients globally suffer from epilepsy, making it the utmost public psychoneurological disorder, according to the WHO (Feigin et al. 2025). The central nervous system (CNS) is modulated by the inhibitory neurotransmitter gamma-aminobutyric acid (GABA), which influences certain processes like anxiety, cognition, sedation/sleep, and convulsions. Furthermore, GABA can stop neuronal excitement and preserve the inhibitory tone. Seizures may occur when there is an imbalance between excitatory and inhibitory neurotransmission (Johnson 2019).

The primary approach to pharmacologically treating epilepsy and convulsions is still to normalize or improve GABAergic inhibition through suppression of GABA neuronal reuptake and degradation, and allosteric regulation of GABA receptors (Khazipov 2016). In addition, the inhibition of synaptic excitation stimulated by glutamate receptors represents another important target for anti-epileptic drugs (Chen et al. 2023). Moreover, it has been extensively deduced that CNS dysfunction arises during epilepsy linked to oxidative insult to the brain tissues, consequently neuroinflammation response (Parsons et al. 2022).

Even though there have been many studies on the pharmacotherapy of epilepsy, only roughly 65% to 70% of patients can achieve good control of seizures with a rather broad spectrum of antiepileptic medications (AEDs) with varying modes of action, including sustained action (Gesche et al. 2020). Surprisingly, various serious adverse effects are reported to be related to the AEDs including a rise in attack frequency, and the decline of encephalographic markers (Vossler et al. 2018). Therefore, searching for novel safe agents that show promise in managing epilepsy and convulsions is of a global interest.

Plants and plant-derived products offer promising candidates for management of several illnesses (Deabes et al. 2025; Esmail et al. 2024; Fahmy et al. 2025). The tropical plant *Lagerstroemia loudonii* Teijsm & Bin (Lythraceae) is mostly found in northern Asia as a wild plant and cultivated worldwide as ornamental tree. *L. loudonii* barks were used in traditional Thai herbal medicine to treat conditions like diarrhea and diabetes, as well as diuretics (Boonphong 2013). Dichloromethane and methanol extracts from *L. loudonii* were shown to possess anti-TB, anti-malarial, and antioxidant properties (Boonphong et al. 2003). The phytochemical screening of *L. loudonii* leaves displayed traces of alkaloids while flavonoids were abundantly present. Moreover, steroids were also found (Boonphong 2013). Otherwise, there is no comprehensive research previously performed on this plant organ from the chemical perspective.

Nonetheless, current developments in nanotechnology allow developing of innovative vehicles and techniques that present extremely intriguing therapeutic prospects for many disorders including CNS by enhancing bioavailability (Baraka et al. 2023; El-Abd et al. 2025; Mowaad et al. 2025; Ruiz and Castro 2016). Nanosuspensions showed the potential to enhance the cerebral effect of the active ingredient (Kamel et al. 2018). Stabilizers are indispensable for the production of nanosuspensions, as they play a pivotal role in inhibiting particle accumulation. The most prevalent stabilizing techniques are steric and electrostatic approaches (George and Ghosh 2013). The electrostatic method is attained by adsorbing ionic surfactants, such as repulsion between particles (Tang et al. 2013). On the other hand, to accomplish steric stabilization, nonionic surfactants such as Poloxamers or polymers like β-cyclodextrin (β-CD) are adsorbed onto the particle surface creating a steric barrier that prevents particle coalescence.

Among these stabilizers, β-CD and its derivatives stand out due to their affordability, non-toxic characteristics, great water solubility, and ability to enhance drug solubility and reduce interfacial tension, making it a potential candidate for stabilizing nanosuspensions (de Freitas et al. 2012). Particle-stabilized nanosystems were found to offer a good and successful approach as drug carriers (AbouSamra et al. 2022; Ghanem et al. 2024; Kamel et al. 2021a). This method provides several advantages, such as improved safety, environmental friendliness, and reduced carcinogenicity by avoiding the use of a large amount of surfactants. Cyclic oligosaccharides known as cyclodextrins (or CDs) are created when starch is broken down by enzymatic reaction, their characteristic structure can enable the enhanced solubility and permeability of molecules (Loftsson and Duchene 2007).

This study affords deep insight into the diversity of phytoconstituents of *L. loudonii* leaf extract (LLE) utilizing LC–ESI–MS/MS in dual negative and positive modes for the first time to our knowledge. This study is the first to scientifically evaluate its neuroprotective properties, highlighting its novelty in the field of epilepsy research. The identification of diverse bioactive compounds, particularly phenolics, further underscores the plant’s therapeutic promise and supports its potential role as a source of novel anticonvulsant agent.

Moreover, in order to enhance the bioavailability and biological potential of the extract, the nanoencapsulation approach has been used to prepare the CD-stabilized nanosuspensions-loaded LLE. Further, the anticonvulsant activity of both LLE and the nanosuspension was assessed against maximum electric shock (MES) or pentylenetetrazole (PTZ)-induced acute seizures in mice. In addition, the effects of LLE and nanosuspension have been evaluated against the oxidative and nitrosative stresses, disturbances in neurotransmitters, GABA depletion, and neuroinflammation that involved in the progression of epileptic seizures.

## Materials and methods

### Phytochemical studies

#### Chemicals

All chemicals used were of high laboratory grade, purchased from Merck (Germany) and Sigma (USA).

#### Collection and preparation of *L. loudonii* leaves

The leaves of *L. loudonii* were gathered from a garden located in Giza, Egypt, and the plant was kindly authenticated by Mrs. Treasa Labib (Consultant of plant taxonomy, Ministry of Agriculture, Egypt). The dried powdered leaves of *L. loudonii* (Teijsm & Bin), 500 g, were successively extracted using 70% ethanol. The rotary evaporator (Heidolph, Germany) was used to concentrate the extracted material that was kept in the refrigerator till use.

#### Characterization and identification of phytochemical constituents of *L. loudonii* leaf extract

##### UPLC–ESI-QTOF–MS/MS analysis

Ultra-performance liquid chromatography in conjunction with electrospray ionization quadrupole time-of-flight tandem mass spectrometry in both negative and positive modes was used to conduct the metabolomics analysis of the total extract.

The following conditions were met: ExionLC (High flow LC) separation by chromatography. Acquisition of TripleTOF 5600+ (Sciex) IDA. Analysis of data: MS-DIAL. The precolumn was Phenomenex in-line filter disks, measuring 0.5 μm by 3.0 mm. The column was 2.1 × 150 mm, 2.5 μm, X choose HSS T3 (Waters). The mobile system was: mobile phase A (1% methanol in a 5 mM ammonium formate buffer at pH 3,) mobile phase B (5 mM ammonium formate buffer with 1% methanol at pH 8) and mobile phase C (Acetonitrile 100%). Rate of flow was 0.3 mL/min. 40 °C was the column temperature. Injection volume was10 µL. 28 min was the run time. Software called Analyst TF 7.1.1 was used to see the compounds’ peaks.

### In vitro free radical scavenging capability

Using the stable DPPH (2,2-diphenyl-1-picrylhydrazyl, Sigma, USA) assay (Hwang and Do Thi 2014), the free radical scavenging potential of the crude extract was ascertained, where the DPPH concentration was 50 μm. A blank of pure methanol was used to measure the absorbance at 517 nm (A) after 60 min. Using a calibration curve made with Trolox, the antioxidant activity was calculated and reported as milligrams of Trolox equivalent (TE) per gram of material.

### Nanoencapsulation study

#### Materials

Methylated-beta-cyclodextrin or Kleptose Crysmeb^®^ (MβCD) was kindly provided by Roquette, Lesterm, France. Poloxamer 407 (P407) was purchased from Sigma Aldrich, St. Louis, MO, USA.

#### Methods

##### Preparation of the extract-loaded MβCD-stabilized nanosuspension (MβCD-NS)

The extract was dispersed in an aqueous solution, then the dissolved P407 (1% w/w) and MβCD (1–5% w/w) were transferred to the aqueous dispersion of the extract and mixed at 300 rpm for 30 min. The mixture was then subjected to homogenization for 2 min at 20,000 rpm using Heidolph Homogenizer, Schwabach, Germany. The concentration of P407 in the final preparation was 1% w/w, that of MβCD was ranging from 1 to 5% w/w and that of the extract was 20 mg/ml. The preparations were then left at room temperature for 24 h without any agitation before visual examination.

##### Sedimentation volume

Sedimentation volume (F) was determined by measuring the height of settled particles after 24 h of static storage at room temperature. Using the following formula, the sedimentation volume was determined as the ratio of the ultimate settled height (Hu) to the initial height (Ho) (Jansook et al. 2020):

$$F=Hu/Ho$$

To test the long term physical stability, the selected formula was then stored for 18 months at room temperature or in the refrigerator at 5 °C (Zulbeari and Holm 2025) and re-examined.

##### Particle size analysis

A Zetasizer (Malvern Instrument, Worcestershire, UK) was used to estimate the particle size, size distribution, and zeta potential of the chosen formulation. Following appropriate dilution, measurements were made at 25 °C and a 90° scattering angle.

##### Enacpsulation efficiency

After preparation, the nanosuspension was separated according to the previously described method (Abbas and Kamel 2020; Kamel et al. 2016, 2022c; Salama et al. 2016). Then, the nanoparticles were sonicated for 30 min. in ethanol, and the solution was filtered with a 0.45 μm syringe filter (Millex-LG, Millipore Co., USA). The active agents were analyzed spectrophotometrically at 280 nm based on previous studies (Ghanem et al. 2024; Kurkin et al. 2020; Sammani et al. 2021) using Shimadzu UV spectrophotometer, 2401/PC, Japan.

Finally, encapsulation efficiency was calculated using the following equation:

$$EE=(Amount\;of\;encapsulated\;drug/Total\;drug\;amount)\;\times\;100$$

##### In-vitro release study

The release kinetics of the active agent from the nanosuspension were determined using the dialysis bag diffusion method and compared to the release from a standard aqueous extract suspension containing an equivalent dose. Samples were sealed within a pre-soaked cellulose dialysis bag (MWCO 12,000–14,000). Release was conducted in a 70% alcoholic aqueous medium (Domínguez-Villegas et al. 2014; Ghanem et al. 2024; Kamel et al. 2021b) agitated at 50 rpm at 37 ± 0.5 °C in a shaking water bath. During the study, aliquots were periodically sampled from the medium and replaced with fresh medium. The amount of released active agent was quantified at 280 nm using a UV spectrophotometer (Ghanem et al. 2024; Kurkin et al. 2020; Sammani et al. 2021) and the cumulative percentage release curves were generated.

##### Transmission electron microscopy

The morphological features of the chosen preparation were determined using Transmission electron microscopy (TEM) (JEM-1230, JEOL, Akishima, Japan). The chosen mixture was diluted 1:10 in distilled water and applied to carbon-coated copper grids. A 2% (w/w) phosphotungstic acid solution was utilized to negatively stain the grids.

### Pharmacological studies

#### Materials

Carbamazepine (Tegretol^®^) was purchased from Novartis Company, Egypt. PTZ was from Sigma, USA, and phenytoin was acquired from ACDIMA International Company, Arab Caps, Obour City, Egypt.

#### Animals and ethics

Male Swiss albino mice (22–26 g) used in the current investigation, were procured from the animal house colony at National Research Centre, Giza, Egypt. Under appropriate conditions of temperature of 22 ± 3 °C and 12/12 h light/dark cycle, the animals were placed in Plexiglas cages with free access to water and food (Basal mice pellets). The study was carried out following the ARRIVE guidelines and instructions established by Ethical Conduct in the Care and Use of animals, and approved by the Medical Ethical Committee of National Research Centre (MERC, Giza, Egypt) under approval number 01420924.

#### Acute toxicity study

In accordance with the OECD 425 standards (Guideline 2001); a total of 18 male mice were used in this study, where the mice were randomly and equally divided into three groups (Normal, LLE, and MβCD-NS). Per group, the mice were then split into two groups of three each. Three mice were orally administered a dose of 2000 mg/kg bw of LLE or MβCD-NS at the beginning of the trial. Four hours prior to the testing substance being administered, the animals were fasted. Meanwhile, for normal group, the mice were orally administered the vehicles of the test substances. Following treatment, the mice were denied food for two hours and their mortality was monitored. The remaining three mice were given the same dosage of the tested materials because there was no mortality. For the first four hours after dosage, and then at regular intervals for the next 14 days, the tested animals were monitored for death, behavioral changes, and clinical indicators of toxicity. At the 15th day, the mice were anesthetized using isoflurane inhalation and the blood samples were collected by heart puncture. Then, the serum was separated for assessing liver and kidney function tests including (alanine aminotransferase (ALT), albumin, and creatinine) using commercial kits supplied by Biodiagnostic company, Giza, Egypt. At autopsy, liver and kidney tissues were isolated and kept in 10% formalin solution for the histopathological evaluations.

Two dosages of LLE or MβCD-NS (equivalent to 100 mg extract/kg/day for the low dose and 200 mg extract/kg/day for the high dose) were chosen for oral administration in this investigation.

#### Experimental protocols of acute seizures models

Both the acute MES and PTZ seizure models were employed to comprehensively assess the anticonvulsant potential of LLE and MβCD-NS. The MES model is a well-established paradigm for evaluating compounds effective against generalized tonic–clonic seizures, primarily through their ability to inhibit seizure spread via voltage-gated sodium channel blockade. In contrast, the PTZ-induced seizure model reflects absence and myoclonic seizure activity, which is closely linked to disturbances in GABAergic neurotransmission. The combined use of these two complementary models allows for a broader evaluation of anticonvulsant efficacy across distinct mechanistic pathways.

##### MES-induced seizures

The mice were randomly sectioned into 6 groups (*n* = 8) as follows: Group1 (Normal Control); mice were administered vehicles. Groups 2 and 3 (LLE 100 and LLE 200), in which, the mice were treated with LLE at the doses of 100 and 200 mg/kg bw/orally, respectively. Groups 4 and 5 (MβCD-NS 100 and MβCD-NS 200), the mice were treated with MβCD-NS at doses equivalent to 100 and 200 mg/kg bw/orally, respectively. Group 6 (Reference group); the mice were given phenytoin (25 mg/kg bw, i.p) (Aghamiri et al. 2020).

The cornea was treated with tetracaine ophthalmic solution (0.5% tetracaine hydrochloride) for local anesthesia and 0.9% saline to improve electrical conductivity, precisely one hour following the prescribed therapy. Using an electrical source for mice, “Ugo Basil, ECT Unit, 57,800 adjusted to a fixed 35 mA current intensity and 3 s shock duration, and at 50 Hz,” electric-induced convulsions have been administered by ear-attached conductors (Aghamiri et al. 2020). Following the electrical stimulation, each animal was watched separately for 30 min in a plastic cage with an open top. Given that the test chemical may inhibit tonic hind limb extension for 180 degrees, it may be able to slow the spread of seizures brought on by MES. Using the survival rate and the previously given equation, the percentage protection is computed (Chrościńska-Krawczyk et al. 2016).

$$\%Protection=(Number\;of\;mice\;protected/Total\;number\;of\;mice)\ast100$$

##### PTZ-induced seizures

The mice were randomly sectioned into 6 groups (*n* = 10) as follows: Group1 (Normal Control); mice were administered vehicles. Group 2 (PTZ); the mice were administered PTZ and vehicles for a week. Group 3 and 4 (LLE 100 and LLE 200), in which, mice were treated with LLE at doses of 100 and 200 mg/kg bw/orally for 7 consecutive days, respectively. Groups 5 and 6 (MβCD-NS 100 and MβCD-NS 200), in which, the mice were treated with MβCD-NS at doses of 100 and 200 mg/kg bw/orally for 7 consecutive days, respectively. Group 7 (Carbamazepine); the mice were given an intraperitoneal injection of Carbamazepine at a dose of 100 mg/kg bw for a week (Anwar et al. 2025).

All experimental animals received intraperitoneal administration of PTZ (85 mg/kg) on the seventh day, one hour after the assigned treatment (Khazaei et al. 2019). Following injection, convulsive behavior was monitored for 30 min, and the ensuing seizures were graded using the following system: **score 0** – no response; **score 1** – trembling behavior arrest; **score 2** – sudden arrest and immobile staring; **score 3** – facial twitching and neck jerks; **score 4** – clonic seizure while seated; **score 5** – clonic seizure without loss of equilibrium; **score 6** – tonic–clonic seizures; **score 7** – tonic–clonic falls on one side; **score 8** – uncontrolled jumping; and **score 9-**tonic extension of forelimbs and hindlimbs (severe tonic seizure) **score 10** – tonic extension leading to death (Van Erum et al. 2019).

At the end of the experiment, the mice were subjected to behavioral tasks. After that, the mice were sacrificed under anesthesia using isoflurane inhalation and the brain tissues were dissected to the assessment of oxidative stress-related parameters as well as the neurotransmitters. Sections of brains were harvested and preserved in 10% formalin solution for the histopathological and immunohistochemical evaluations.

#### Behavioral assessments

##### Locomotor activity test

Each mouse in the tested groups had their spontaneous motor activity recorded by the Activity cage (M 7420; Ugo Basile, Italy) at the end of the experiment. Every mouse had one hour to acclimate to room temperature on the day of the test session. The activity was measured using the conventional infrared photocell approach, which records any disruption. A total of the photocell interferences was recorded for four minutes (Baraka et al. 2020).

##### Rotarod test

By an accelerating Rotarod (Model No. 7750; Ugo Basile, Italy), the mice’s motor coordination was evaluated in accordance with the protocol outlined by Vijitruth et al. (Vijitruth et al. 2006). For three days in a row, mice received three training sessions. To achieve a steady performance, all mice were skilled on the Rotarod apparatus at a set speed of 4 rotations per minute (rpm). The mice were put on the testing rod during the test session, and the Rotarod’s speed gradually increased from 4 rpm to 40 rpm over the course of 4 min. Each mouse’s falling latency time was then documented.

#### Biochemical analyses

##### Determination of lipid peroxidation biomarker

 Production of MDA standard solution: a volume of 25 μL of 1,1,3,3 tetraethoxypropane (TEP) was dissolved in 100 ml of water to create the MDA standard (1mmol). By hydrolyzing 1 milliliter of TEP stock solution in 50 milliliters of 1% sulfuric acid and letting it sit at room temperature for two hours, the working standard was created. A serial dilution of MDA standard (20-1.25 nmol/ml) was done (Karatepe, 2004). 

The samples were examined using HPLC (Agilent HP 1100 series, USA) with Supelcosil C18 analytical column (5 μm particle and 80 Å pore size) (250 × 4.6 ID). The mobile phase was set at a flow rate of 1.5 ml/min at 250 nm. It contains 30 mmol KH_2_PO_4_ and methanol (65%–35%, H3PO4 by pH 4) (Karatas et al. 2002).

##### Determination of nitric oxide

Using the method of Papadoyannis et al. (Papadoyannis et al. 1999), the amount of nitric oxide (NO) in brain tissues was measured by HPLC (Agilent HP 1100 series, USA) as nitrate and nitrite. 150 × 4.1 mm, 10 μm anion exchange PRP-X100 Hamilton was used as the analytical column. Methanol and 0.1 M NaCl were combined in a 45:55 volume ratio to form the mobile phase, with a wavelength of 230 nm and a flow rate of 2 mL/min.

With a stock concentration of 1 mg/ml, sodium nitrite and sodium nitrate were utilized for the reference standard production. To ascertain the retention periods and peak separation, a typical nitrite and nitrate mixture was employed. In the combination solution, the amounts of nitrite and nitrate were equal.

##### Determination of glutathione (oxidized and reduced)

By employing the Jayatilleke and Shaw approach, HPLC was employed to identify the thiol molecules of reduced and oxidized glutathione (GSH and GSSG, respectively) (Jayatilleke and Shaw 1993). The reference standards for GSSG and GSH were acquired from Sigma Chemical Co. diluted and dissolved in 75% methanol in stock at a concentration of 1 mg/ml before HPLC application.

The chromatogram and report were taken from the Agilent-purchased Chemstation application. At a wavelength of 210 nm and a flow rate of 2 milliliters per minute, Synerji RP Max column 3.9 was employed. Acetonitrile at pH 2.7 was employed as an isocratic mobile phase in potassium phosphate buffer.

##### Determination of brain HO-1, and IL-6

The brain tissues were homogenized using a tissue homogenizer purchased from Biospec Product, mini-BeadBeater-8, USA, to create a 10% (w/v) tissue homogenate in a 0.05 M phosphate buffer (pH 7). To exclude cell debris, nuclei, erythrocytes, intact cells, and mitochondria, the homogenate was centrifuged at 10,000 rpm for 20 min at 4 °C using a cooling centrifuge (Laborezentrifugen, 2k15, Sigma, Germany). For the acquired supernatants were separated to estimate the content of heme-oxygenase 1 (HO-1), and interleukin 6 (IL-6) using a mouse ELISA kit supplied by Fine Test^®^ (Catalogue No: EM1128, Wuhan, China) and Biolegend (Catalogue No: 431301, Switzerland). Bradford’s method was used to quantify the brain homogenate’s total protein concentration using a kit from Genei, Bangalore.

##### Determination of brain monoamines

Using solid phase extraction CHROMABOND column NH_2_ phase (Cat. No. 730031), the sample was promptly separated from the lipids and trace elements. Then, the sample was directly injected into an AQUA column 150 mm 5µ C18 (Phenomenex, USA) at pH 2.5, flow rate 1.5 ml/min, UV 190 nm, and mobile phase 20 mM potassium phosphate. After 12 min, serotonin (5HT), dopamine (DA), and norepinephrine (NE) were separated.

Every monoamine concentration in the sample was detected by the ensuing chromatogram in comparison to the standard, and the content was ultimately determined as µg per gram brain tissue (Pagel et al. 2000).

##### Determination of brain GABA and glutamate

Using the phenylisothiocyanate (PITC) derivatization process, HPLC (Agilent HP, USA, 9 × 30 cm PICO-TAG column, Waters) was used to determine the amount of GABA, and glutamate (GLU) in the brain tissues (Heinrikson and Meredith 1984). Eluent (1) and Eluent (2) were Triethylamine, and PITC respectively.

The following were the assay conditions: Wavelength: 250 nm; temperature: 46 °C; flow rate: 2 ml/min. The sample tissue was weighed and homogenized in 1/10 weight/volume of 75% aqueous HPLC grade methanol as the initial step in the HPLC technique.

The process of derivatization began by drying the test sample using a drying solution made up of a 2:2:1 (by volume) mixture of methanol, 1 M sodium acetate trihydrate, and triethylamine (TEA). After thoroughly shaking the dry sample with the drying solution, it was placed under vacuum until it was completely dry. After giving the dried sample a good shake and letting it stand at room temperature for 20 min, the derivatizing solution was added and vacuumed until it was completely dry. After that, a sample diluent made of 0.71 g of disodium-hydrogen phosphate and 10% phosphoric acid was used to dilute the dry sample at pH of 7.4.

The resultant solution was then combined with 5% acetonitrile. For HPLC separation, the derivatized samples and standards were introduced into the column in 20 µl volumes. Each amino acid in the sample was determined by the chromatogram as µg/gram brain tissue.

#### Histopathological studies

##### Hematoxylin and Eosin

The samples (Six samples per group) preserved in neutral buffered formalin (10%) were fixed in paraffin after dehydration step. The paraffin blocks were partitioned at 5 micron thickness, and then stained by Hematoxylin and Eosin stain (Bancroft and Gamble 2008).

##### Histopathological lesion score

The brain’s histopathological alterations were categorized as follows: mild (less than 30%), moderate (30%−50%), and severe (more than 50%) (El-Maksoud et al. 2020). These alterations were given a score ranges from 0 to 3, where 0, 1, 2, and 3 means no, mild, moderate, and severe changes, respectively.

##### Immunohistochemistry

In accordance with the instructions given by Shamseldean et al. (Shamseldean et al. 2022), the immunohistochemistry method was accomplished. Tissue samples were rehydrated in different alcohol grades after being deparaffinized in xylene. The antigen retrieval was performed by pretreating the sections for 20 min with a pH 6 citrate buffer. Anti-TNF alpha antibody [TNF/1500R] (ab270264; 1:100 dilution rate, Abcam, Cambridge, UK) and Anti-Nrf2 antibody [EP1808Y] (ab62352; 1:100 dilution rate, Abcam, Cambridge, UK) were applied to sections and incubated for two hours in a humidified chamber. Goat anti-rabbit IgG H&L (HRP) (ab205718; Abcam, Cambridge, UK) was used to incubate the sections in addition to 3,3′-diaminobenzidine tetrahydrochloride (DAB, Sigma) as a chromogen. The slides were mounted using DPX, and the counterstain was hematoxylin. On the negative control slides, primary antibodies were substituted with PBS.

##### Quantitative evaluation of TNF-α and Nrf2 immunostaining

The quantitative immunoreactivity of TNF-α and Nrf2 was evaluated according to a previous study (Shaaban et al. 2024). A high-power (x 400) microscopic field was used to investigate the immuno-reactivity in 5 fields per section. Color deconvolution picture J 1.52 p software (Wayne Rasband, National Institutes of Health, USA) was used to estimate the percentage of positively stained cells (%).

### Statistical studies

The sample size was determined using G-Power software (version 3.1.9.4; Faul et al., Germany). The primary outcome measure was predefined as the reduction in brain γ-aminobutyric acid (GABA) levels, a major inhibitory neurotransmitter implicated in epileptogenesis. Reduced GABA concentration results in continuous cortical neuronal excitation, leading to convulsive episodes resembling human seizures. Based on previously reported data demonstrating a significant difference in brain GABA levels between pentylenetetrazole (PTZ)-induced epileptic and control groups (Koshal and Kumar 2016), the sample size was calculated to detect an effect size of 0.876 with a study power of 95% (1 – β error probability) and a two-tailed α of 0.05, applying a continuity correction. Accordingly, six animals per group were deemed sufficient; however, to compensate for potential mortality during the experimental period, ten animals were included in each group. Hence, for most endpoints, group sizes of *n* = 6 were used except score clonic convulsions, *n* = 10.

Furthermore, the study was designed as a controlled, randomized experimental study. Animals were randomly assigned to experimental groups using a computer-generated random sequence. Allocation concealment was maintained by having a researcher not involved in interventions or outcome assessments perform the group assignments. Outcome assessments were performed in a blinded manner by investigators unaware of group allocation.

The data’s normal distribution was confirmed by the Shapiro-Wilk’s test for normality (*p* > 0.05). The statistical difference between means was ascertained using one-way analysis of variance (ANOVA). The statistical significance between various groups was assessed using Tukey’s test for multiple comparisons. The statistical analyses for the in vivo investigation were performed with GraphPad Prism (GraphPad Inc., San Diego, CA; version 8.0 for Windows).

## Results

### Phytochemical results

#### UPLC-ESI–QTOF-MS/MS analysis

UPLC/MS is the most recent chromatographic analytical tool for metabolites identification in a natural field, here we give a spot on metabolic different classes in *L. loudonii* leaves crude extract as a novelty, the identification of compounds was attained by matching our spectral data with available literature and the database published on online software: FOODB, HMDB& Mass bank. In our study, dual acquisition modes “positive and negative” were utilized to cover a wide range of metabolites having different polarities. UPLC as a reversed-phase chromatography, the sequence of compounds elution was ordered from the most polar to the least.

**Fig. 1:**
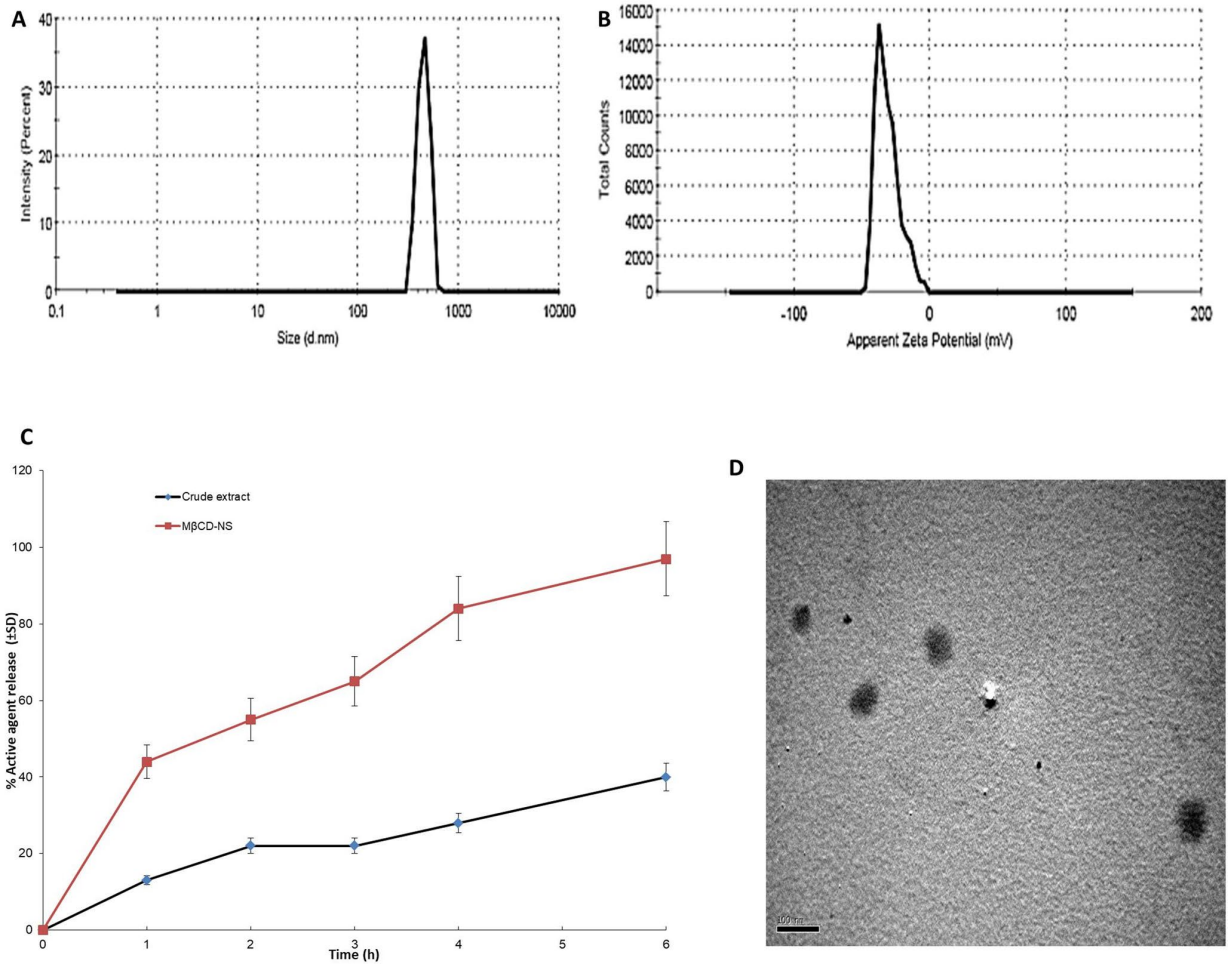
(**A**) Particle size, (**B**), Zeta potential value, and (**D**) microphotograph of the selected MβCD-NS. (**C**) In-vitro release profiles. MβCD-NS; extract-loaded MβCD-stabilized nanosuspension

**Fig. 2:**
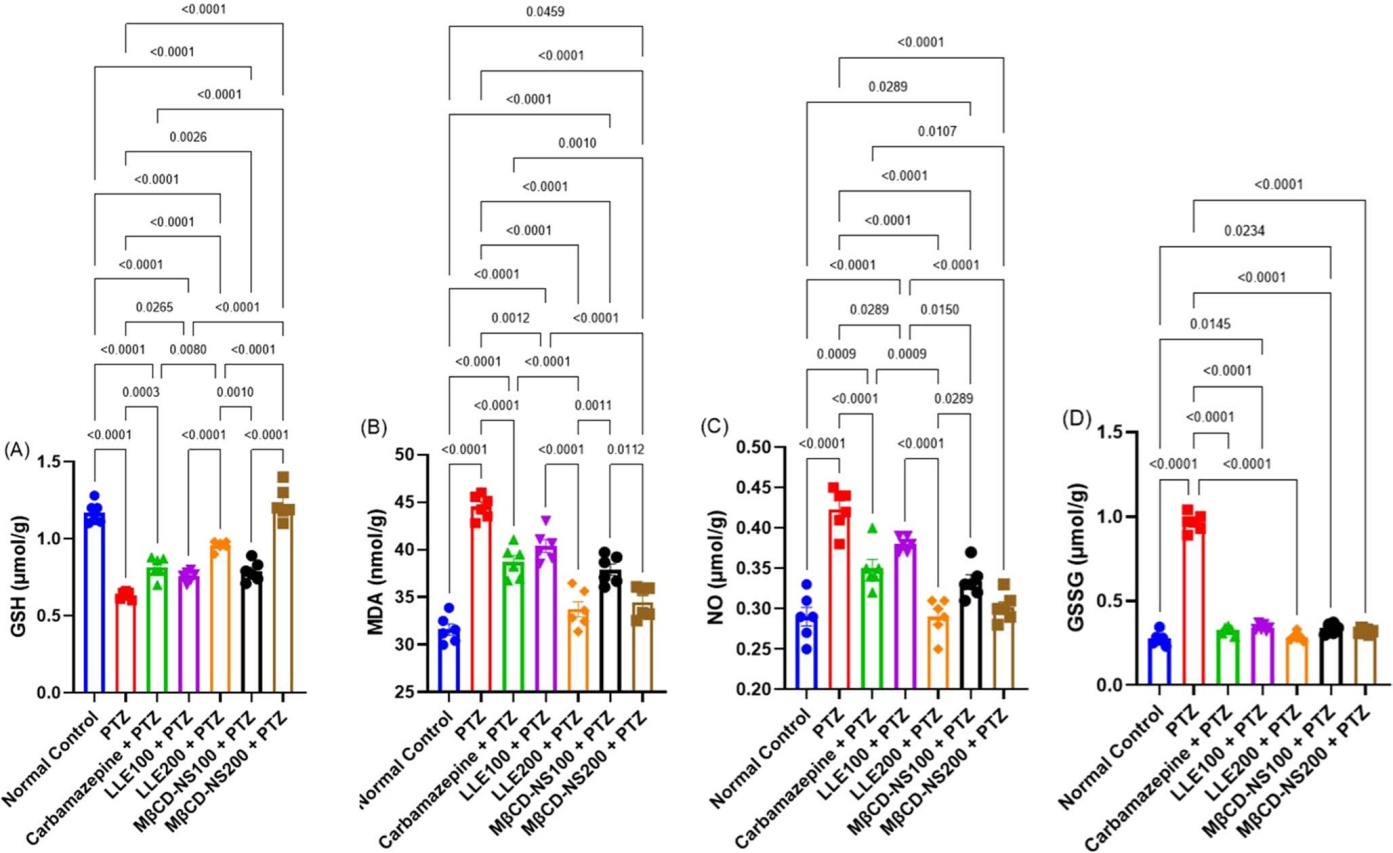
Effects of LLE or MβCD-NS on the changes in brain oxidative stress biomarkers; GSH (**A**), MDA (**B**), NO (**C**), and GSSG (**D**) in PTZ-induced convulsions in mice. Data are expressed as Mean ± SE (*n* = 6) and analyzed by one-way ANOVA followed by Tukey´s multiple comparison test. PTZ; pentylenetetrazole, LLE; ethanolic leaf extract of *Lagerstroemia loudonii*, MβCD-NS; extract-loaded MβCD-stabilized nanosuspension, GSH; glutathione, MDA; malondialdehyde, NO; nitric oxide, GSSG; oxidized glutathione

In our study, 57 compounds from negative and positive annotations were identified (Table 1; Figs. 1S and 2S), 47 of them have not been reported before from the species of interest. Metabolites were categorized to different classes: 17 flavonoids including flavonoid -*O*- hexosides, *C*-hexosides and methoxylated flavonoids, 8 phenolic acid derivatives, 6 coumarins, 3 sugars, 5 nitrogenous compounds, 10 fatty acids only two of them are saturated, 4 triterpene derivatives, 3 organic acids and one anthraquinone (trihydroxy-dimethoxy-methyl anthraquinone).

**Table 1:** LC–ESI–MS/MS analysis of *L. loudenii* leaf extract

No.	Rt	[M-H]^−^/[M + H]^+^	M. formula	Error	Fragmentation	Compound name
Organic acids						
1.	1.05	133.0136	C_4_H_5_O_5_^−^	9.3	115, 87	Malic acid
2.	1.06	191.0189	C_7_H_11_O_6_^−^	−1.8	173, 85, 59	Quinic acid
3.	5.52	265.1435	C_15_H_21_O_4_^+^	−7.4	265, 229, 219	Abscisic acid
Phenolic acids						
4.	1.39	153.0245	C_7_H_5_O_4_^−^	9.2	109, 108	Dihydroxybenzoic acid
5.	1.44	193.0355	C_10_H_9_O_4_^−^	9.4	149, 134	Ferulic acid
6.	2.38	137.0237	C_7_H_5_O_3_^−^	9.1	93	*p*-Hydroxybenzoic acid
7.	3.41	341.0889	C_15_H_17_O_9_^−^	−1.1	179,161,135	Caffeic acid hexoside
8.	4.01	355.1024	C_16_H_19_O_9_^+^	0.7	193, 191, 163, 135	Chlorogenic acid
9.	5.37	301.0028	C_14_H_5_O_8_^−^	−9.0	282, 242	Ellagic acid
10.	5.99	315.0167	C_15_H_7_O_8_^−^	−3.6	301, 299	Methylellagic acid
11.	8.24	207.0654	C_11_H_11_O_4_^−^	6.9	177, 149, 121, 105	Sinapoyl aldehyde
Sugars						
12.	1.12	195.0514	C_6_H_11_O_7_^−^	4.5	177, 159, 141	Gluconate
13.	1.31	341.1107	C_12_H_21_O_11_^−^	3.1	179, 161, 143	Gentiobiose
14.	1.39	181.0709	C_6_H_13_O_6_^−^	5.4	163, 112, 101, 71	Galactitol
Nitrogenous compounds						
15.	1.30	116.0691		18.8	70	Proline
16.	1.36	96.04376	C_5_H_6_NO^+^	20	78, 70, 68	3-Hydroxypyridine
17.	1.70	140.0338		4	122, 112, 94, 76, 66	6-Hydroxynicotinic acid
18.	1.72	154.0495	C_7_H_8_NO_3_^+^	0.8	108, 136, 80	3-Hydroxyanthranilic acid
19.	3.17	144.0498	C_6_H_10_NOS^+^	−2.2	144, 126, 113, 112, 84, 70	4-Methyl-5-thiazole ethanol
Coumarins						
20.	2.96	339.0736	C_15_H_15_O_9_^−^	4.4	177, 133, 121	Esculin
21.	4.47	193.0495		5.4	193, 175, 163, 151, 133	Scopoletin
22.	7.76	179.0703	C_10_H_11_O_3_^+^	−7.1	151, 137, 121	Mellein
23.	10.25	177.0546	C_10_H_9_O_3_^+^	6.7	162, 133	Hymecromone
24.	11.91	207.0642	C_11_H_11_O_4_^+^	−4.2	192,164,149, 121	Dimethylcoumarin
25.	12.77	217.0495	C_12_H_9_O_4_^+^	5.6	202, 174	Methoxypsoralen (methoxyfurocoumarin)
Flavonoids						
26.	6.27	431.0979	C_21_H1_9_O_10_^−^	5.8	341, 311	Kaempferol-C-rhamnoside
27.	6.31	609.1450	C_27_H_29_O_16_^−^	−1.5	447, 285	Luteolin-3’, 7-di-O- hexoside
28.	6.54	463.0892	C_21_H_19_O_12_^−^	−4.6	301	Isoquercetin
29.	6.78	447.0922	C_21_H_19_O_11_^−^	4.6	357, 327	Isoorientin
30.	6.83	593.1501	C_27_H_29_O_15_^−^	0.7	447, 431, 285	Kaempferol-7-neohesperidoside
31.	7.41	609.1818	C_28_H_33_O_15_^+^	6.5	463, 447, 301	Diosmin
32.	8.98	593.1290	C_30_H_25_O_13_^−^	2.1	447, 431, 285	Kaempferol-3-O-hexoside-2’’-p-coumaroyl
33.	9.02	609.1257	C_30_H_25_O_14_^−^	−1	447, 285	Luteolin 7-(2’’-p-coumaroylglucoside
34.	9.09	489.1745	C_23_H_21_O_12_^−^	−14.9	327, 300, 285, 151	Kaempferol-3-O-acetyl-hexoside
35.	9.41	285.0406	C_15_H_9_O_6_^−^	2.5	151, 133	Luteolin
36.	10.46	447.09	C_21_H_19_O_11_^−^	−3.4	429, 357, 327	Luteolin-6-C- hexoside
37.	10.58	269.0451	C_15_H_9_O_5_^−^	3.5	151	Apigenin
38.	11.09	315.0507	C_16_H_11_O_7_^−^	−0.6	301, 297, 255, 247,151	3’-Methoxy-4’,5,7-trihydroxyflavonol (*Isorhamnetin)*
39.	11.12	301.0701	C_16_H_13_O_6_^+^	−6.3	286, 285, 229, 153	Methoxy-dihydroxy flavone
40.	12.74	343.1176	C_19_H_19_O_6_^+^	−1.9	328, 313, 299, 283, 282	Tetramethoxy flavone
41.	14.17	313.1071	C_18_H_17_O_5_^+^	−1.4	298, 297, 270, 269, 255	Trimethoxy flavone
42.	16.39	419.1337	C_21_H_23_O_9_^+^	−10.3	404,389, 374, 371, 328	Hydroxy-hexamethoxyflavone
Fatty acids						
43.	11.85	293.2126	C_18_H_29_O_3_^−^	−1.9	275, 249, 113	Hydroxy-octadecatrienoic acid
44.	12.56	295.2269	C_18_H_31_O_3_^−^	−3.7	277, 195, 183,	Hydroxy-octadecadienoic acid
45.	14.25	297.2451	C_18_H_33_O_3_^−^	2.57	275, 183	Hydroxy-octadecenoic acid
46.	16.47	313.2387	C_18_H_33_O_4_^−^	2.97	174, 185	Dihydroxy-octadecenoic acid
47.	19.40	277.2176	C_18_H_29_O_2_^−^	3.4	---	Octadecatrienoic acid
48.	20.35	279.2327	C_18_H_31_O_2_^−^	7.7	---	Linolenic acid
49.	20.74	253.2172	C_16_H_29_O_2_^−^	6.3	----	Hexadecenoic acid
50.	21.35	281.2449	C_18_H_33_O_2_^−^	−5.8	281, 112	Octadecenoic acid
51.	24.12	255.2324	C_16_H_31_O_2_^−^	5	237	Palmitic acid
52.	24.14	283.2632	C_18_H_35_O_2_^−^	8.1	----	Stearic acid
Triterpene derivatives						
53.	13.8	577.2280	C_30_H_41_O_11_^−^	−9.2	499, 354, 288, 218, 112	Triterpene derivative
54.	20.16	415.3207	C_27_H_43_O_3_^+^	−9.2	272, 271, 253	Diosgenin
55.	22.21	455.3531	C_30_H_47_O_3_^−^	2	409, 391, 318, 255	Oleanolic acid
56.	22.26	455.3518	C_30_H_47_O_3_^−^	−10.7	409, 391, 318	Ursolic acid
Anthraquinone						
57.	13.19	329.0661	C_17_H_13_O_7_^−^	−1.0	314, 313, 299	Trihydroxy-dimethoxy-methyl anthraquinone

*Rt* retention time

Table 1 annotated the identified compounds, three organic acids were recorded, malic acid appeared at *m/z* 133.0136 (C_4_H_5_O_5_^−^) and abscisic acid at *m/z* 265.1435 (C_15_H_21_O_4_^+^) showing products ions at 87& 219, respectively corresponding to loss of CO₂ (−44 *amu*) followed by H₂ (2 Da) (Shakour et al. 2023).

Regarding phenolic acids, 4 hydroxycinnamic acid derivatives were identified: chlorogenic acid (peak 8) was detected at *m/z* 355.1024 (C_16_H_19_O_9_^+^) confirmed from fragment ion appeared at *m/z* 191 corresponding to quinic acid that detected in our platform (peak 3). Caffeic acid hexoside (peak 7) appeared at *m/z* 341.0889 (C_15_H_17_O_9_^−^) and gave the following ions at *m/z* 179 [M-H- hexose] ^−^, 161 [M- H- hexose-18] ^−^, 135 [M-H- hexose- COO]^−^. Both compounds’ fragmentations were in accordance with Zheleva-Dimitrova et al. (Zheleva-Dimitrova et al. 2017). Ferulic acid (C_10_H_9_O_4_^−^) was detected at *m/z* 193.0355 and sinapoyl aldehyde (peak 11) was predicted at *m/z* 207.0654 (C_11_H_11_O_4_^−^) and gave distinctive peak at *m/z* 177 for [M-H-CHO-2 H]^−^. Additionally, 4 hydroxybenzoic acid derivatives were detected; ellagic acid and its methyl derivative (peaks 9 & 10) at *m/z* 301.0028& 315.0167 [C_14_H_5_O_8_& C_15_H_7_O_8_]^−^, respectively with mass difference 15 *amu* for methyl group, in addition to *p*-hydroxy benzoic acid and its dihydroxylated derivative (peaks 1&6).

Furthermore, peaks from (26–42) represented 17 flavonoid compounds belonging to different subclasses; 11 flavones and 6 flavonols categorized as: 6 O-glycosides, 3 C- glycosides, 2 aglycones and 6 methoxylated flavonoids. Sequence of O-glycosides fragmentation started by losing the sugar molecule, viz., [M-H-162]^−^ appeared when losing a hexose moiety, while [M-H-146]^−^ assigned for rhamnose loss as showed in peaks (27, 28, 30&32–34). Moreover, C-glycosides (peaks 26, 29& 36), undergo cross-ring cleavage (0,2 and 0,3) giving two distinctive peaks at *m/z* [M-H-90] ^−^ and [M-H-120]^−^ for (C_3_H_6_O_30_^−^) and (C_4_H_8_O_4_^−^) loss, respectively (Farag et al. 2019). For the 6 methoxylated flavonoids, they were identified in the positive ionization mode (peaks: 31& 38–42) except for isorhamentin (peak 38). They were recognized by demthylation (−15 *amu*) and demethoxylation (−30 *amu*). Moreover, two flavone aglycones were detected (peaks 35& 37), and identified as luteolin at *m/z* (C_15_H_9_O_6_^−^) and apigenin (C_15_H_9_O_5_^−^).

Ellagic acid, methylellagic acid, kaempferol-rhamnoside and kaempferol-3-O-acetyl-hexoside were previously detected from other *Lagerstromia* species rather than our plant of interest (Kim et al. 2020). While Kaempferol-3-O-hexoside-2’’-p-coumaroyl is first detected in the current study from *Lagerstromia.* Morever, our previous work on other species; ”*Lagerstromia* indica” proved the presence of *p* -hydroxy benzoic, chlorogenic, ferulic and ellagic acids in the total leaf extract (Elsawi et al. 2018).

On the other hand, 6 coumarins were annotated at peaks (20–25). All were identified from positive mode except for esculin (dihydroxycoumarin hexoside) from negative mode. It was found at *m/z* 339.0736 [C_15_H_15_O_9_]^−^ giving fragments at *m/z* 177 [M-H-hexose] ^−^ and 133 [M-H-hexose- COO]^−^. Moreover, scopoletin (6-methoxy-7 hydroxycoumarin) detected at *m/z* 193.0495 [C_10_H_9_O_4_]^+^ showed the characteristic fragments at *m/z* 175 and 163 corresponding to dehydration and demethoxylation, respectively. Two other related coumarins with mass difference 2 *amu*, hydroxy-methyl coumarin (hymecromone) and dihydro-hydroxy-methyl-isocoumarin (mellein) detected at *m/z* 177.0546 [C_10_H_9_O_3_]^+^ and 179.0703 [C_10_H_11_O_3_]^+^, correspondingly. Methoxypsoralen (methoxyfurocoumarin) appeared at *m/z* 217.0495 [C_12_H_9_O_4_]^+^ and provided product ions at *m/z* 202 [M + H-CH_3_]^+^ and 174 [M + H- C_2_H_3_O] ^+^. It is noteworthy that coumarins were detected from other *Lagerstromia* species while they were identified for the first time from *L. loudonii* (Dou Hui et al. 2005; Zhao et al. 2009).

Ten fatty acids have been identified (peaks 43–52), categorized as 8 unsaturated; four of them were hydroxylated (peaks 43–46); and 2 saturated fatty acids were recorded (peaks 51&52). Hydroxy-octadecatrienoic, hydroxy-octadecadienoic, hydroxy-octadecenoic, dihydroxy-octadecenoic, octadecatrienoic acids (peaks 43–46) and oleic acid (peak 50) have been detected from *L. loudonii* leaves for the first time in the current study. While palmitic and linolenic acids were reported before from *L. indica* and *L. speciose* leaves (Sirikhansaeng et al. 2017). The presence of a hydroxyl group in hydroxy fatty acids has drawn interest to their biological properties (Zhang et al. 2024). It is to be noted that fatty acid fragmentation pattern was compatible with that in literature (Farag et al. 2014; Kim et al. 2020).

Four triterpene derivatives (peaks 53–56) have been identified (Table 1). Oleanolic and ursolic acids were previously isolated from *L. loudonii* fruits (Boonphong 2013), but first identified herein from the plant leaves, their fragmentations matched that aforementioned in literature (Kim et al. 2020).

### In vitro antioxidant activity

The data obtained from the DPPH assay informed that the antioxidant activity of the extract is 322.64 ± 0.40 mgTE/g.

### Characterization of the prepared MβCD-NS

#### Visual and physical examination of the prepared MβCD-NS

**Fig. 3:**
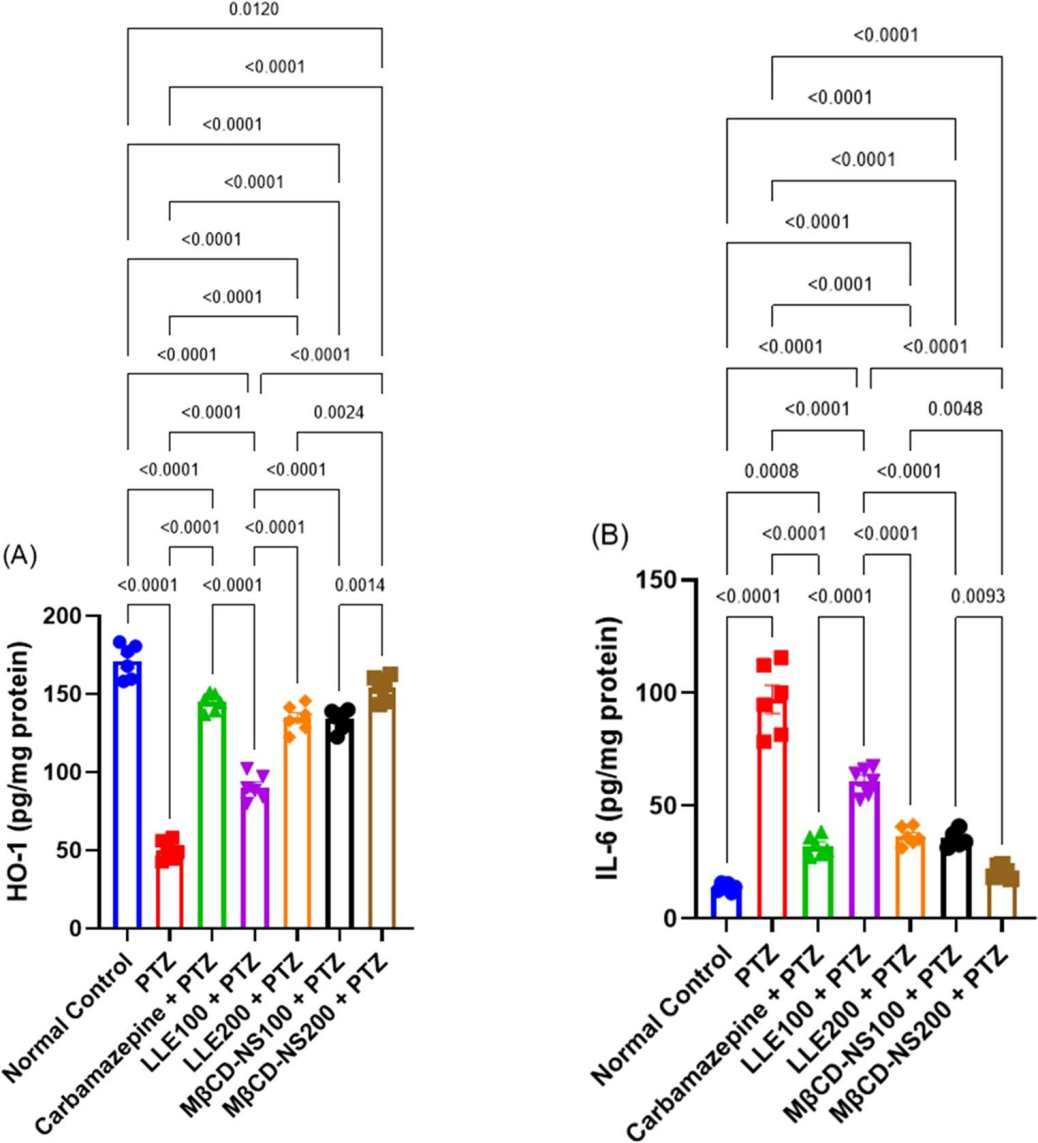
Effects of LLE or MβCD-NS on the changes in brain IL-6, and HO-1 in PTZ-induced convulsions in mice. Data are expressed as Mean ± SE (*n* = 6) and analyzed by one-way ANOVA followed by Tukey´s multiple comparison test. PTZ; pentylenetetrazole, LLE; ethanolic leaf extract of *Lagerstroemia loudonii*, MβCD-NS; extract-loaded MβCD-stabilized nanosuspension, IL-6; interleukin 6, HO-1; hemeoxygenase 1

The photos of the prepared MβCD-NS after storage for 24 h are displayed in Fig. 3S. In case of the MβCD-NS prepared using a concentration of 1 and 2% MβCD, settling of solid phase can be easily seen; while at a concentration of 5% MβCD the nanosuspension was uniform and no sediment was formed.

The sedimentation volume (F) was calculated and was found to be 0.60 ± 0.05, 0.65 ± 0.05 and 1.00 ± 0.00 for the nanosuspensions prepared using 1, 2 and 5 MβCD %, respectively. The value of F can range from 0 to 1. Generally, a higher F value correlates with greater product stability. When F reaches 1, no sediment is apparent and caking is prevented. Therefore, the nanosuspension stabilized using 5% MβCD was selected for further investigation.

During the storage period at room temperature or in the refrigerator at 5 °C (18 months), no change can be observed in the selected nanosuspension. The sedimentation volume (F) value didn’t change, which can indicate the long-term physical stability.

#### Particle size analysis and encapsulation efficiency

The particle size of the selected MβCD-NS was found to be 445.8 ± 61.08 nm (Fig. 1A). It was previously reported that the nanoencapsulation of active ingredients, of herbal source, with low aqueous solubility can increase their solubility and membrane permeability which can allow a better absorption and oral bioavailability (AbouSamra et al. 2023; Kamel 2021; Kamel et al. 2020, 2022a).

The PDI was found to be equal to 0.298 ± 0.02; such a small value indicates the homogeneity and uniformity of the nanosuspension under study.

The Zeta potential value was found to be −31.2 ± 8.65 mV (Fig. 1B) indicating the physical stability and low tendency for aggregation of the prepared nanosuspension, as it was reported that charged particles with high zeta potential values (≥|30| mV) exhibit reduced particle aggregation due to electrostatic repulsion.

The encapsulation efficiency of the selected MβCD-NS was found to be equal to 83.25% ± 2.48.

Figure 1C is showing the release profile of the active agents from the prepared nanosuspension compared to the crude extract, the solubilizing and release enhancing effect of the formulated MβCD-NS is clear, as it attained an almost complete release at about 6 h while, that of the crude extract was only about 40% (p ˂0.05).

#### Transmission electron microscopy (TEM)

The microphotographs of the MβCD-NS under study are in shown in Fig. 1D. Well-separated, almost spherical nanoparticles can be seen. However, the particle size detected by the TEM was lower than that detected DLS technique, this was previously reported and attributed to the shrinkage of the particles during preparation for electron microscopy imaging (Dubes et al. 2003; Kamel et al. 2022b).

### Pharmacological and biochemical evaluations

#### Acute toxicity study

**Fig. 4:**
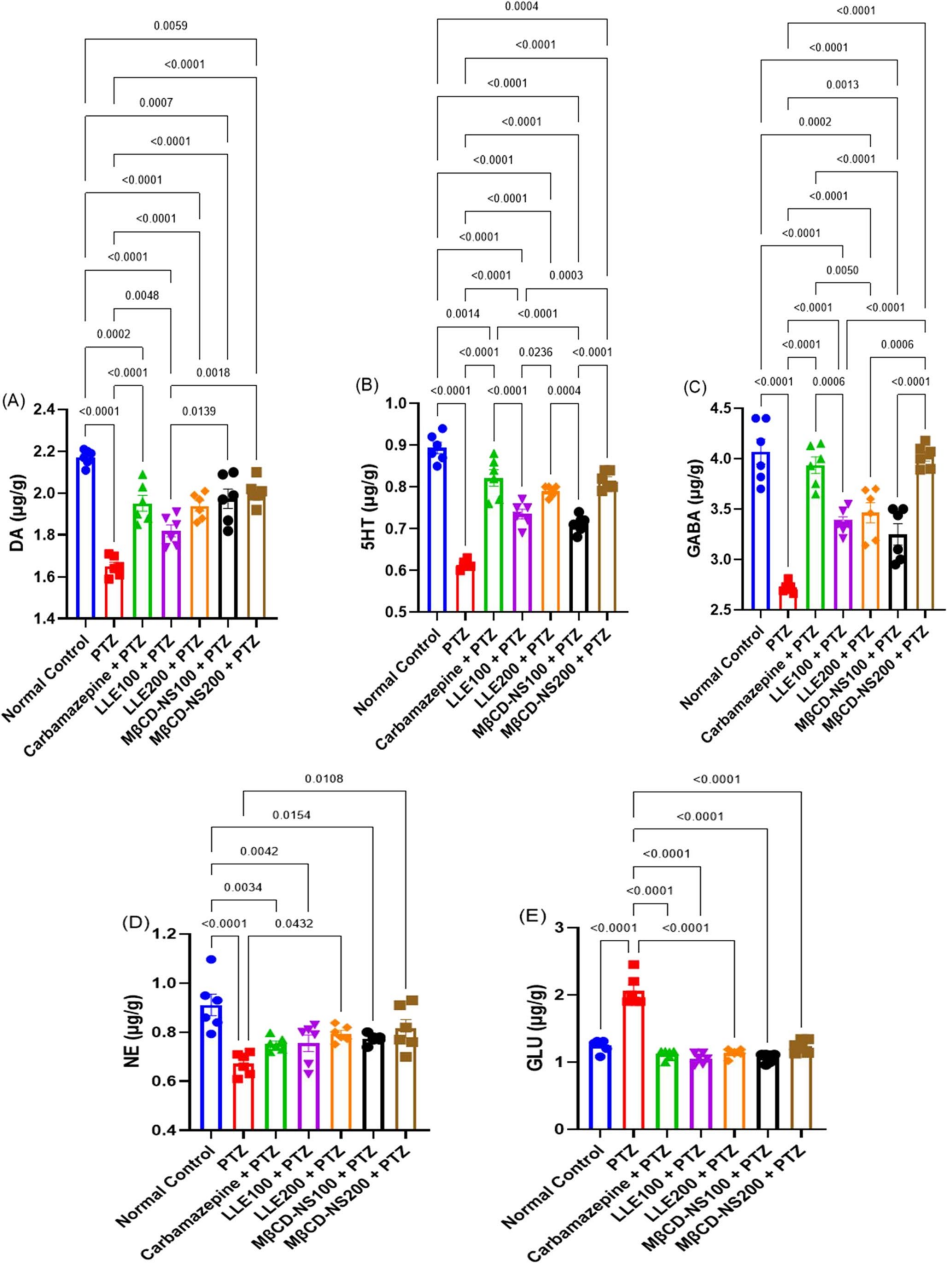
Effects of LLE or MβCD-NS on changes in the brain neurotransmitters; DA (**A**), 5HT (**B**), GABA (**C**), NE (**D**), and GLU (**E**) in PTZ-induced convulsions in mice. Data are expressed as Mean ± SE (*n* = 6) and analyzed by one-way ANOVA followed by Tukey´s multiple comparison test. PTZ; pentylenetetrazole, LLE; ethanolic leaf extract of *Lagerstroemia loudonii*, MβCD-NS; extract-loaded MβCD-stabilized nanosuspension, NE; norepinephrine, 5HT; serotonin, DA; dopamine, GABA; gamma-aminobutyric acid, GLU, glutamate

The results of the acute toxicity documented that both LLE and MβCD-NS were safe up to the dose of 2 g/kg bw. Notably, no changes in serum ALT, AST, albumin, urea, and creatinine levels were noticed when compared to the normal mice (Table 2). In addition, the histopathological investigations showed normal hepatic and renal tissues of mice treated with LLE or MβCD-NS similar to those of the normal group (Fig. 4S, Supplementary file). Moreover, there were no recorded toxic symptoms of the treated animals; hence the extract and its prepared nanosuspension were safe at the adopted dose levels.

**Table 2:** Effect of LLE and MβCD-NS on serum hepatic and renal function biomarkers in mice

Groups/parameter	Normal	LLE	MβCD-NS
ALT (U/L)	35.28 ± 0.51	36.05 ± 0.81	36.81 ± 1.29
AST (U/L)	71.61 ± 1.99	74.97 ± 2.02	73.88 ± 1.97
Albumin (g/dL)	2.62 ± 0.04	2.61 ± 0.03	2.70 ± 0.03
Creatinine (mg/dL)	0.80 ± 0.02	0.82 ± 0.03	0.77 ± 0.018
Urea (mg/dL)	21.91 ± 0.99	21.68 ± 1.05	22.45 ± 1.21

*ALT* alanine aminotransferase, *LLE* ethanolic leaf extract of *Lagerstroemia loudonii*, *MβCD-NS* extract-loaded MβCD-stabilized nanosuspension

#### Effects of LLE and MβCD-NS on percentage protection in maximum electric shock and score of clonic convulsions, and locomotor activity in PTZ-induced seizures in mice

As presented in Table 3, the oral administration of both LLE and MβCD-NS at the two dose levels significantly protected the mice from the onset of seizures induced by the action of maximum electric shock, when compared to the untreated mice. Furthermore, compared to the PTZ-intoxicated mice, a significant improvement was recorded in reducing the severity of convulsions in the mice treated with LLE or MβCD-NS at doses of 100 and 200 mg/kg. In addition, the mice locomotor activity dramatically decreased in PTZ group as indicated by a significant reduction in falling latency time and activity counts in mice, in respect to the normal animals. In contrast, both LLE and MβCD-NS significantly preserved the mice’s locomotor activity, compared to the positive epileptic group. It be noted that, among the treated groups, MβCD-NS (200 mg/kg bw) manifested the best records concerning MES or PTZ-induced convulsions models.

**Table 3:** Effects of LLE and MβCD-NS on percentage protection in MES and score of clonic convulsions, and locomotor activity in PTZ-induced seizures in mice

Groups	% Protected 35 mA (Mortality rate) (*n* = 8)	Score of clonic convulsions (Mortality rate) (*n* = 10)	Falling latency time (Sec) (*n* = 6)	Activity counts (*n* = 6)
	MES- induced seizures	PTZ-induced seizures		
PTZ	-----	9.1 ± 0.28^*#^ (40%)	14.00 ± 2.35^*#^	4.5 ± 0.76^*#^
LLE100	75 (0%)	1.2 ± 0.38 ^@#^ (20%)	100.00 ± 6.02*^@#^	98.83 ± 6.38*^@#^
LLE200	87.5 (0%)	0.9 ± 0.23 ^@^ (0%)	232.33 ± 4.81^@^	128.33 ± 7.67^@^
MβCD-NS 100	87.5 (0%)	1.1 ± 0.31^@^ (0%)	146.50 ± 3.13*^@#^	98.50 ± 2.70*^@#^
MβCD-NS 200	100 (0%)	0.8 ± 0.2^@^ (0%)	230.5 ± 3.20^@^	121.5 ± 6.03^@^
Phenytoin	100 (0%)	-----	-----	-----
Carbamazepine	-----	1.1 ± 0.23^@^ (10%)	238.17 ± 1.22^@^	120.33 ± 3.08^@^
Normal control	0 (50%)	0 ± 0.00^@^ (0%)	240.00 ± 0.00^@^	131.33 ± 3.35^@^

Data are expressed as Mean ± SE, and analyzed by one-way ANOVA followed by Tukey´s multiple comparison test at *p* < 0.05. As compared to (*) normal control, (^@^) PTZ positive control and (#) reference drug (Carbamazepine)

*PTZ* pentylenetetrazole, *LLE* ethanolic leaf extract of *Lagerstroemia loudonii*, *MβCD-NS* extract-loaded MβCD-stabilized nanosuspension

####  Effects of LLE or MβCD-NS on changes in brain oxidative stress and inflammation biomarkers

A profound incidence of oxidative stress condition has been documented in the mice of PTZ-group, as demonstrated by a significant reduction in the brain GSH, and HO-1 content, along with a boost in MDA, GSSG, and NO levels in brain tissues at *p* < 0.001 when compared to those of the normal control mice. Concerning the decline in GSH, and HO-1 content following PTZ administration, treatment with LLE at the dose of 200 mg/kg or MβCD-NS at the two adopted doses markedly improved its levels in the brain tissues. Moreover, a significant decline in the MDA, GSSG, and NO levels has been observed in the LLE and MβCD-NS-treated groups relative to the PTZ-intoxicated animals, reflecting the powerful antioxidant capacity of LLE and its nanosuspension with superior action of the MβCD-NS at the high dose level. Similar results were noted for these parameters in the Carbamazepine + PTZ-treated mice (Figs. 2 and 3A).

Similarly, a significant rise (*p* < 0.0001) in the inflammatory marker; IL-6 was depicted in the brain tissue of PTZ-treated group, maximizing the occurrence of inflammatory response. Meanwhile, marked reductions in the levels of IL-6 were documented in the brain tissues of rats treated with LLE and MβCD-NS, compared to the PTZ-group. It is worth mentioned that the oral administration of MβCD-NS at the two dose levels showed higher anti-inflammatory properties than the corresponding dose of LLE (Fig. 3B).

####  Effects of LLE or MβCD-NS on changes in the brain neurotransmitters

As compared to the normal records of the control group, a marked disturbance in the brain neurotransmitters has been documented in the PTZ-epileptic group, as indicated by a significant reduction in the neuronal NE, DA, 5HT, and GABA contents, as well as elevation of GLU level. These changes were reversed by LLE or MβCD-NS dose-dependently. It’s worth mentioning that MβCD-NS at the high dose level succeeded in normalizing or preserving the NE, DA, and GABA levels in the brain tissues when matched to the normal group, deducing its potential neuroprotective action. The results of LLE and MβCD-NS have been statistically compared to those of Carbamazepine as depicted in Fig. 4.

#### Histopathological findings

##### Hippocampus

**Fig. 5:**
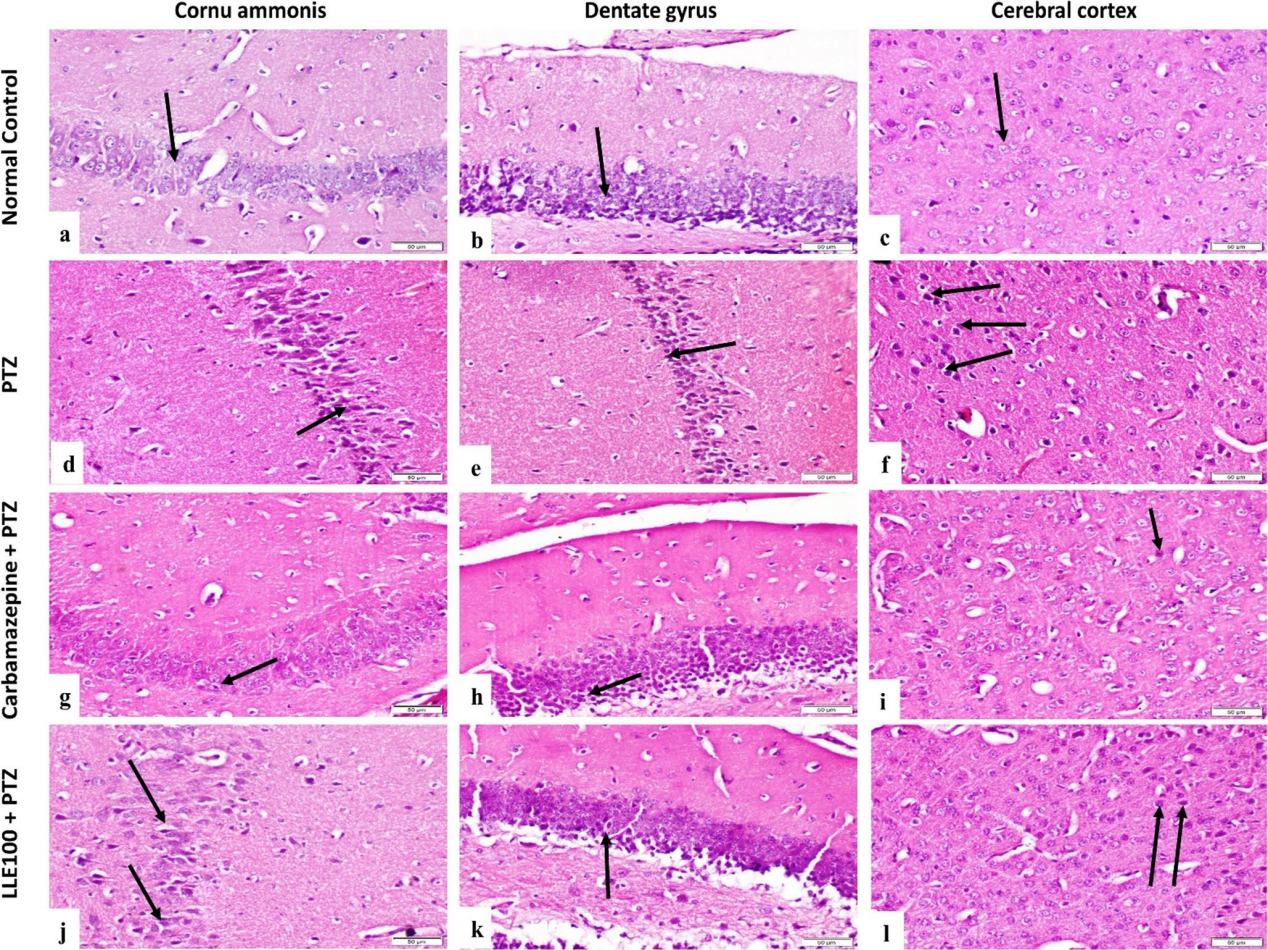
Photomicrographs of mice hippocampus and cerebral cortex, (H&E stained sections, scale bar 50 μm). **(a)**,** (b) & (c)** normal control group showing normal histological structure of CA, DG and cerebral cortex respectively (arrow). **(d)** PTZ group showing decreased cell population of CA with massive neuronal degeneration (arrow). **(e)** PTZ group showing decreased cell population of DG and massive neuronal degeneration (arrow). **(f)** Cerebral cortex of PTZ group showing neuronal degeneration of considerable number of neurons (arrows). **(g)** & **(h)** Carbamazepine + PTZ group showing few degenerated neurons in CA and DG (arrow). **(i)** Cerebral cortex of Carbamazepine + PTZ group showing few degenerated neurons (arrow). **(j) & (k)** CA and DG of LLE100 + PTZ group showing degeneration of some neurons (arrows). **(l)** LLE100 + PTZ group cerebral cortex showing moderate neuronal degeneration (arrows), CA: cornu ammonis, DG: dentate gyrus. PTZ; pentylenetetrazole, LLE; ethanolic leaf extract of *Lagerstroemia loudonii*, MβCD-NS; extract-loaded MβCD-stabilized nanosuspension

**Fig. 6:**
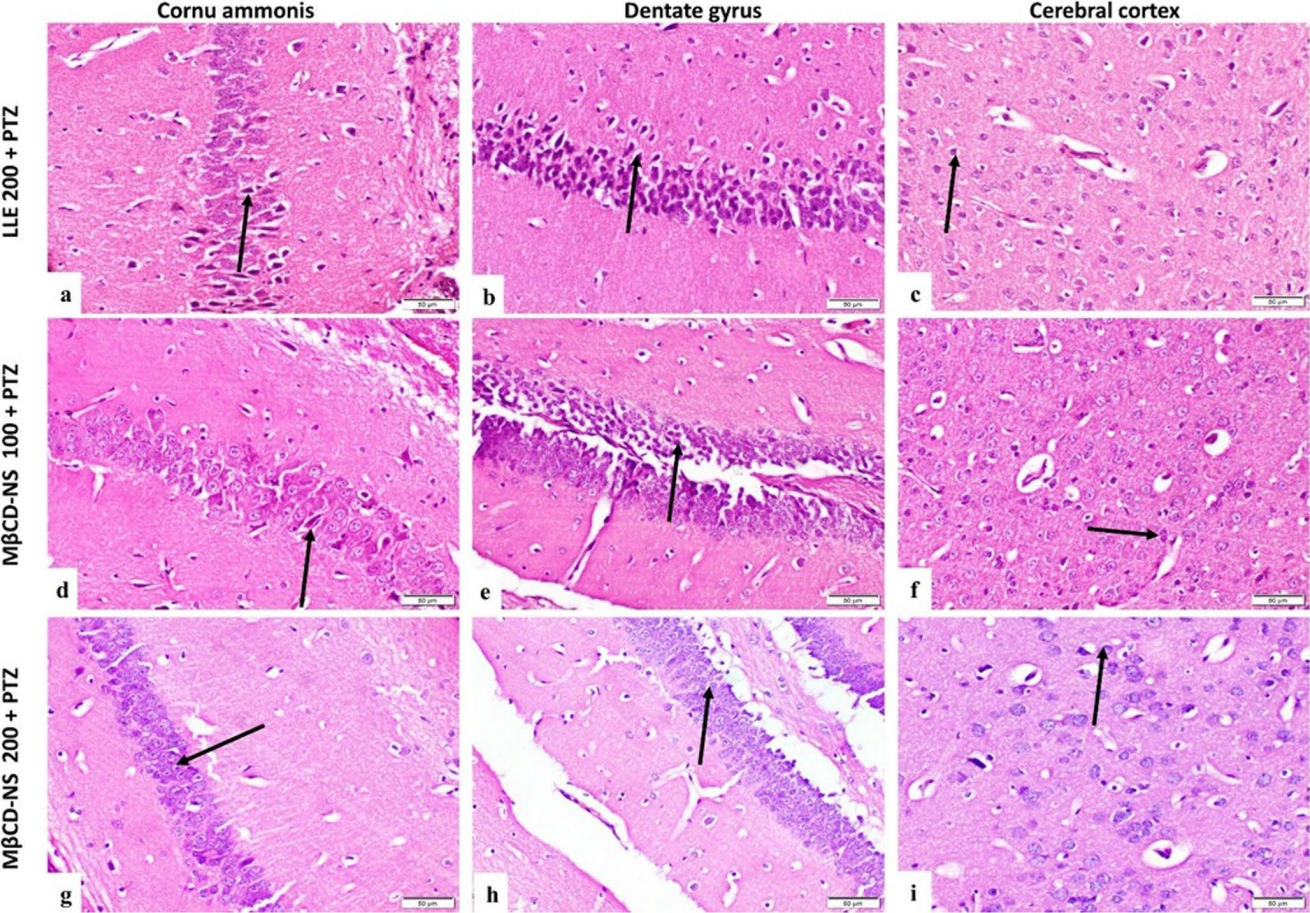
Photomicrographs of mice hippocampus and cerebral cortex, (H&E stained sections, scale bar 50 μm). **(a) & (b)** LLE200 + PTZ group CA&DC showing few degenerated neurons (arrow). **(c)** cerebral cortex of LLE200 + PTZ group showing mild neuronal degeneration (arrow). **(d) & (e)** MβCD-NS 100 + PTZ CA&DC showing mild neuronal degeneration (arrow). **(f)** MβCD-NS100 + PTZ group showing mild lesions in neurons of cerebral cortex (arrow). **(g) & (h)** CA&DC of MβCD-NS200 + PTZ group showing nearly normal structure (arrow). **(i)** MβCD-NS200 + PTZ group showing normal cerebral cortex neurons (arrow), CA: cornu ammonis, DG: dentate gyrus. PTZ; pentylenetetrazole, LLE; ethanolic leaf extract of *Lagerstroemia loudonii*, MβCD-NS; extract-loaded MβCD-stabilized nanosuspension

normal control group showed normal histological structure of cornu ammonis (CA) and dentate gyrus (DG) (Fig. 5a & b). PTZ group showed decreased cell population of CA and DG regions with massive neuronal degeneration in both regions (Fig. 5d & e). Carbamazepine + PTZ group showed noticeable amelioration in hippocampus, degenerated neurons in CA and DG were few (Fig. 5g & h). Lesions of hippocampus of LLE100 + PTZ group were moderate (Fig. 5j & k). Significant improvement was noted in LLE 200 + PTZ group when compared with PTZ group, neuronal degeneration in both regions was fewer (Fig. 6a & b). Concerning MβCD-NS 100 + PTZ and MβCD-NS 200 + PTZ groups, lesions of hippocampus of these groups was very mild and even the tissue showed nearly normal structure (Fig. 6d, e, g & h).

##### Cerebral cortex

cerebral cortex of normal control group revealed normal histological structure (Fig. 5c). PTZ group showed neuronal degeneration of considerable number of neurons (Fig. 5f). Carbamazepine + PTZ group revealed few degenerated neurons in cerebral cortex (Fig. 5i). LLE100 + PTZ group showed moderate neuronal degeneration (Fig. 5L). LLE200 + PTZ group showed mild neuronal degeneration in cerebral cortex (Fig. 6c). MβCD-NS 100 + PTZ and MβCD-NS 200 + PTZ groups showed very mild lesions in neurons of cerebral cortex (Fig. 6f & i). Lesions in hippocampus and cerebral cortex were recorded and scored according to their severity (Table 4).

**Table 4:** Scoring of histopathological alterations in hippocampus and cerebral cortex

Lesions	Normal Control	PTZ	Carbamazepine + PTZ	LLE100 + PTZ	LLE200 + PTZ	MβCD-NS 100 + PTZ	MβCD-NS 200 + PTZ
- **Hippocampus**							
Decrease cell population of CA regions	0	3	0	1	1	0	0
Decrease cell population of DG	0	3	0	1	1	0	0
Neuronal degeneration of CA regions	0	3	1	2	1	1	1
Neuronal degeneration of DG	0	3	1	2	1	1	0
**-** **Cerebral cortex**							
Neuronal degeneration in cerebral cortex	0	3	1	2	1	1	1

The score system was designed as: score 0 = absence of the lesion, score 1= (< 30%), score 2= (< 30% – 50%), score 3= (> 50%). (*n* = 6)

*PTZ* pentylenetetrazole, *LLE* ethanolic leaf extract of *Lagerstroemia loudonii*, *MβCD-NS* extract-loaded MβCD-stabilized nanosuspension

#### Immunohistochemical findings of TNF-α and Nrf2

**Fig. 7:**
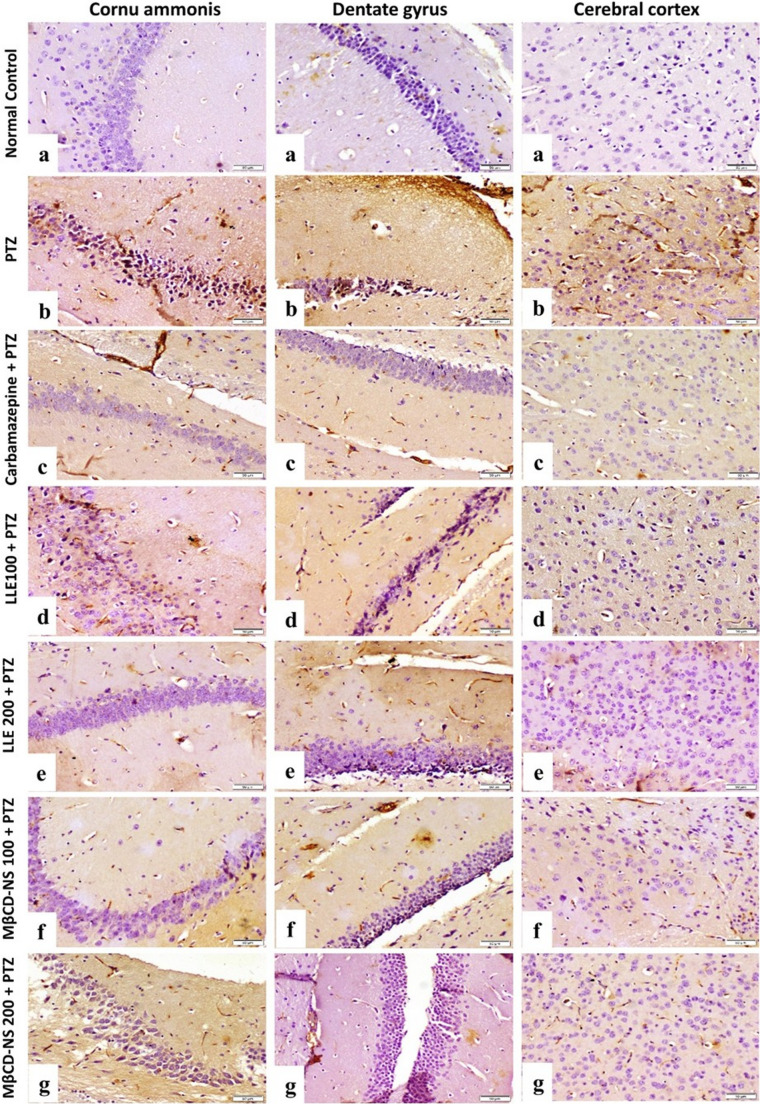
Photomicrographs of mice hippocampus and cerebral cortex (TNF-α stained sections, scale bar 50 μm), **(a)** normal control group showing very weak immune expression, **(b)** PTZ group showing strong immune expression, **(c)** Carbamazepine + PTZ group showing weak immune expression, **(d)** LLE100 + PTZ group showing moderate immune expression, **(e)** LLE200 + PTZ showing mild immune expression, **(f)** MβCD-NS100 + PTZ group showing mild expression of TNF-α **(g)** MβCD-NS200 + PTZ group showing mild expression of TNF-α. PTZ; pentylenetetrazole, LLE; ethanolic leaf extract of *Lagerstroemia loudonii*, MβCD-NS; extract-loaded MβCD-stabilized nanosuspension, TNF-α; tumor necrosis factor α

**Fig. 8:**
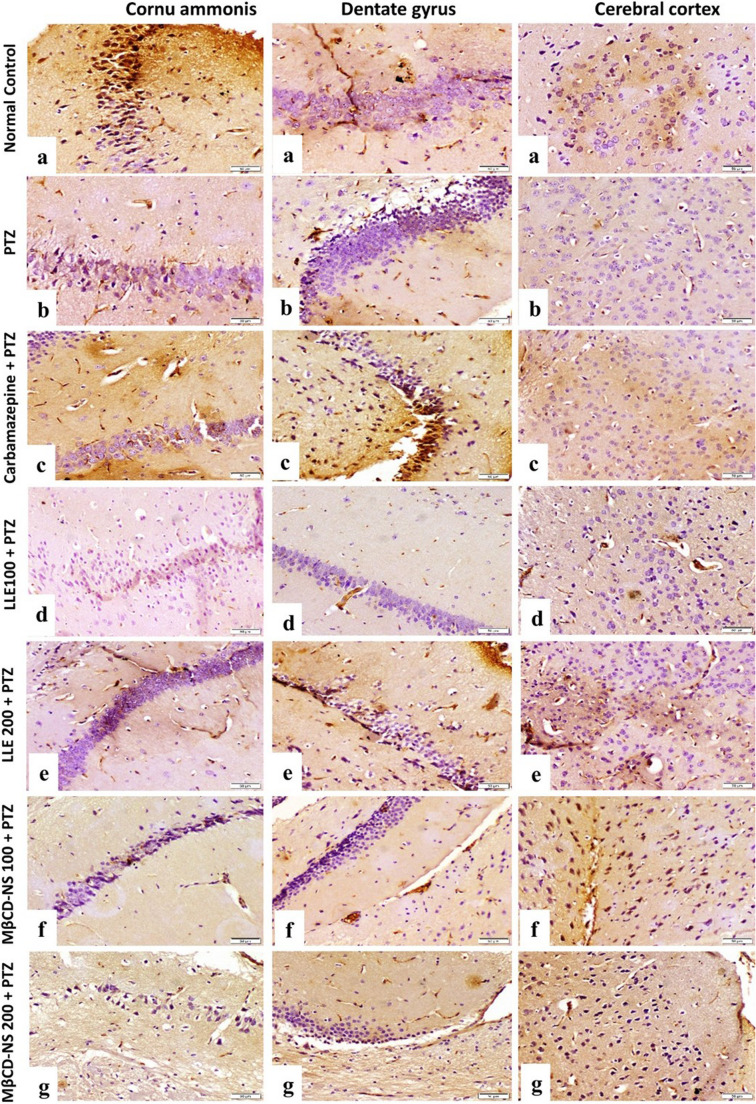
Photomicrographs of mice hippocampus and cerebral cortex (Nrf2 stained sections, scale bar 50 μm), (a) normal control group showing strong expression of Nrf2. **(b)** PTZ group showing weak expression of Nrf2. **(c)** Carbamazepine + PTZ group showing moderate expression of Nrf2. **(d)** LLE100 + PTZ group showing weak to moderate expression of Nrf2. **(e)** LLE200 + PTZ showing moderate expression of Nrf2. **(f)** MβCD-NS100 + PTZ group showing moderate expression of Nrf2 **(g)** MβCD-NS200 + PTZ group showing strong expression of Nrf2. PTZ; pentylenetetrazole, LLE; ethanolic leaf extract of *Lagerstroemia loudonii*, MβCD-NS; extract-loaded MβCD-stabilized nanosuspension, Nrf2; nuclear erythroid related factor 2

Immunostaining expression of TNF-α and Nrf2 area % in brain tissue (CA, DC and cerebral cortex) were illustrated in Table 5. Normal Control group showed very weak immune-expression of TNF-α and strong expression of Nrf2 (Figs. 7a and 8a). PTZ group showed strong immunoreactivity of TNF-α and weak expression of Nrf2 (Figs. 7b and 8b). Carbamazepine + PTZ group showed weak expression of TNF-α and moderate to strong expression of Nrf2 (Figs. 7c and 8c). LLE100 + PTZ group showed moderate expression of TNF-α and weak to moderate expression of Nrf2 (Figs. 7d and 8d). LLE200 + PTZ, MβCD-NS 100 + PTZ, and MβCD-NS 200 + PTZ groups showed mild expression of TNF-α and moderate to strong expression of Nrf2 (Figs. 7e, f & g, and 8 e, f & g, respectively).

**Table 5:** Area % of TNF-α and Nrf2 expression in the different treated groups

Groups	TNF-α Area %			Nrf2 Area %		
	Cornu ammonis	Dentate gyrus	Cerebral Cortex	Cornu ammonis	Dentate gyrus	Cerebral Cortex
Normal Control	4.05 ± 0.25^@#^	1.91 ± 0.15 ^@#^	4.26 ± 0.18^@#^	31.27 ± 1.01^@#^	30.36 ± 0.74^@#^	37.51 ± 0.56^@#^
PTZ	32.47 ± 0.60^*#^	31.21 ± 0.41^*#^	40.49 ± 0.51^*#^	11.94 ± 0.50^*#^	12.39 ± 0.64^*#^	17.14 ± 0.54^*#^
Carbamazepine + PTZ	14.24 ± 0.45^*@^	15.73 ± 0.25^*@^	19.44 ± 0.25^*@^	25.87 ± 0.61^*@^	26.03 ± 0.34^*@^	30.21 ± 0.49^*@^
LLE100 + PTZ	25.53 ± 0.46^*@#^	23.20 ± 0.33^*@#^	28.73 ± 0.42^*@#^	18.03 ± 0.32^*@#^	20.69 ± 0.51^*@#^	20.08 ± 0.26^*@#^
LLE200 + PTZ	19.77 ± 0.37^*@#^	19.19 ± 0.39^*@#^	24.78 ± 0.38^*@#^	22.27 ± 0.59^*@#^	24.18 ± 0.53^*@^	28.58 ± 0.53^*@^
MβCD-NS 100 + PTZ	18.59 ± 0.43^*@#^	16.17 ± 0.33^*@^	21.24 ± 0.58^*@^	26.99 ± 0.49^*@^	26.11 ± 0.46^*@^	29.06 ± 0.49^*@^
MβCD-NS 200 + PTZ	12.29 ± 0.48^*@^	14.04 ± 0.32^*@^	17.34 ± 0.33^*@#^	32.83 ± 0.87 ^@#^	28.59 ± 0.47^@^	33.43 ± 0.73^*@#^

Data are expressed as Mean ± SE (*n* = 5 fields) and analyzed by one-way ANOVA followed by Tukey´s multiple comparison test at *p* < 0.05. As compared to (*) normal control, (^@^) PTZ positive control and (#) reference drug (Carbamazepine). TNF-α; tumor necrosis factor α, Nrf2; nuclear erythroid relator factor 2, PTZ; pentylenetetrazole, LLE; ethanolic leaf extract of *Lagerstroemia loudonii*, MβCD-NS; extract-loaded MβCD-stabilized nanosuspension

## Discussion

Epilepsy is one of the most prevalent and chronic neurological conditions (Beghi 2020). Increased synaptic connectivity of neurons (e.g., excitatory glutaminergic neurons), channelopathies (voltage-gated ion channel shifts, and potassium channel deterioration and/or more persistent sodium channel), disruption of synaptic receptors (e.g., downregulation of GABAergic receptors), elevated excitatory neurotransmission (GLU), and decreased inhibitory neurotransmission (GABA) are documented to be the fundamental and primary mechanisms linked to epilepsy (Wojda et al. 2009).

In this research, the potential anticonvulsant activity of MβCD-NS compared to that of LLE was assessed in Swiss albino mice using different acute seizure models viz. MES, and PTZ-induced seizures. Additionally, behavioral, biochemical, histopathological, and immunohistochemical investigations have been established. These analyses focused on the oxido-inflammatory biomarkers viz. GSH, GSSG, NO, MDA, Nrf2, HO-1, IL-6 and TNF-α, as well as brain neurotransmitters such as NE, DA, 5-HT, GABA, and GLU. This has been coupled with behavioral assessments concerning locomotor activities.

The MES model delivered electroshock in experimental animals produced symptoms that closely resemble those in humans with epilepsy (Aghamiri et al. 2020). The principal mechanism of MES convulsions highlights the effectiveness of agents that block voltage-gated sodium channels such as phenytoin or reduce excitatory amino acid activity and/or counteract their effects to be effective in a model of MES-induced convulsions (Aghamiri et al. 2020). In our study, the MES test validated the potential actions of LLE and its nanosuspension in a dose-dependent manner with the superior protective effect of MβCD-NS (equivalent to 200 mg extract/kg) against electroshock-delivered convulsions in mice. Based on the results obtained in MES-induced seizure test, the LLE and MβCD-NS were further evaluated against PTZ-induced convulsions in Swiss albino mice.

Profound convulsions ranging from hind limb tonic extensor to death have been recorded in PTZ-group, validating the successful establishment of epilepsy model in mice (Van Erum et al. 2019). In contrast, LLE or MβCD-NS significantly reduced the severity of tonic convulsions in the PTZ-challenged mice in a dose-dependent action. Treatment with LLE or MβCD-NS significantly reduced seizure severity in a dose-dependent manner, with MβCD-NS (200 mg/kg) offering the highest protection marked by delayed onset or absence of behavioral symptoms. PTZ-treated mice showed significant motor deficits, as indicated by marked inability of animals’ locomotion along with profound loss of mice’s motor coordination, which agrees with previous research (Nieoczym et al. 2021). In contrast, high-dose LLE and MβCD-NS preserved motor function, as evidenced by increased movement and fall latency, suggesting a neuroprotective effect likely attributed to their bioactive constituents. Oxidative stress plays an integral part in the pathophysiology and progression of seizures (Aguiar et al. 2012). Additionally, the brain’s limited antioxidant defenses, high oxygen demand, and elevated metabolic rate make it particularly vulnerable to oxidative damage, especially during seizures when mitochondrial dysfunction and ROS production intensify neuronal injury (Łukawski and Czuczwar 2023). The brain’s antioxidant defense system, particularly GSH, is crucial for combating oxidative stress, but seizures deplete these protective mechanisms, leaving the brain more vulnerable to further harm (Schmitt et al. 2015). In both human and animal models, epileptic seizures are linked to the diminution of antioxidant enzymes and GSH content in the brain because of the overproduction of ROS and the overconsumption of these enzymes (Wu et al. 2020; Yuan et al. 2020). Additionally, NO, is a chemical linked to the control of epileptic events and neural stimulation capacity through increasing levels of cyclic guanosine monophosphate (cGMP), which in turn boosts GLU activity and exacerbates the epileptic process (Kurt et al. 2016).

The study revealed that PTZ induced a significant oxidative/antioxidative disparity in brain tissue, evidenced by elevated levels of MDA, GSSG, and NO, alongside a marked reduction in GSH compared to the control group. These findings align with previous reports highlighting increased lipid peroxidation and oxidative stress during epileptic seizures. These outcomes confirm earlier findings documented reinforcing the role of oxidative damage in seizure pathogenesis (Alachkar et al. 2020; Tavakoli et al. 2023; Yuan et al. 2020).

Neuroinflammation plays a significant role in the development and progression of epilepsy. Oxidative stress and inflammation often coexist in epileptic patients, suggesting a complex interplay that contributes to epileptogenesis and the onset of seizures (Mukhtar 2024). Experimental models have consistently demonstrated inflammatory responses during seizures (Althagafi 2024; Tavakoli et al. 2023), with pro-inflammatory cytokines interacting with GLU receptors or disrupting GLU metabolism, thereby exacerbating neuronal excitability, underscoring inflammation’s pivotal role in the development of epilepsy (Pernot et al. 2011). The Nrf2 signaling pathway, known for regulating antioxidant defenses including production of antioxidant enzymes like HO-1, has emerged as a promising therapeutic target for both oxidative stress and inflammation (Kaidery et al. 2013). Additionally, previous research have highlighted the anti-inflammatory potential of Nrf2 activation, positioning it as a promising therapeutic strategy for managing neuroinflammatory conditions in various animal models of neurological disorders (Pepe et al. 2018). The concept of the neuroprotective potential of Nrf-2 activators in various animal models has further supported the exploration of Nrf-2 activators as a treatment for neurological disorders (Zgorzynska et al. 2021). In this study, immunohistochemical analysis revealed a significant downregulation of Nrf2, alongside elevated TNF-α expression in the cerebral cortex and hippocampus of PTZ-challenged mice, consistent with previous studies using epilepsy animal models (Campolo et al. 2017; Nkwingwa et al. 2023). In addition, our results documented a marked decline in HO-1 content in the brain tissue of PTZ-treated group, which is in agreement with previous investigations (Hakimi Naeini et al. 2024). Moreover, the incidence of inflammation in neural tissue following PTZ injection was validated by a significant rise in the brain level of IL-6, compared to the normal group, underscoring the role of inflammatory responses in seizure-induced neuronal damage (Ahmad et al. 2025).

Conversely, pre-treatment with LLE or MβCD-NS dose-dependently succeeded to lessen the oxido-inflammatory response in the brain tissues of mice as indicated by our obtained data, compared to the PTZ-group. This was evidenced by a significant decline in NO, GSSG, and MDA along with elevation in GSH levels have been observed in LLE and MβCD-NS-treated groups, maximizing its antioxidant properties as validated by the in vitro DPPH approach of the examined extract. In addition, the immunohistochemical findings revealed the low expression of TNF-α, coupled with high Nrf2 content in the cerebral cortex and hippocampus regions in LLE and MβCD-NS-treated mice. In addition, marked up-regulation in HO-1, and down-regulation in IL-6 levels were documented in the brain tissues of groups treated with in LLE and MβCD-NS. These findings suggest that LLE and MβCD-NS exert notable antioxidant and anti-inflammatory effects, likely attributed to their diverse bioactive phytochemical constituents.

Neurotransmitters such as GABA, GLU, DA, 5HT, and NE play critical roles in the pathophysiology of epilepsy. Numerous local biochemical alterations linked to seizure activity impact the neurotransmitters monoamines and amino acids (Uddin et al. 2018). In the present research, PTZ was observed to lower levels of DA, NE and 5-HT in the brain tissues, when matched to the normal control mice. The findings align with earlier research (Abdel-Rahman et al. 2013; Chimakurthy and Talasila 2010; Visweswari et al. 2010). For epileptogenesis to develop and progress, monoamines are necessary. Similarly, reduced NE levels an endogenous anticonvulsant may be attributed to downregulation of α1 receptors and impaired dopamine-β-hydroxylase activity (Yuan et al. 2020). The decline in 5-HT has been linked to impaired synaptosomal uptake and reduced tryptophan hydroxylase activity (Tchekalarova et al. 2011), while decreased DA levels may result from elevated monoamine oxidase activity and disrupted reuptake mechanisms (Rezaei et al. 2017).Similarly, reduced NE levels an endogenous anticonvulsant may be attributed to downregulation of α1 receptors and impaired dopamine-β-hydroxylase activity (Yuan et al. 2020).

Conspicuously, GABA and GLU are two prominent neurotransmitters among numerous others involved in epileptogenesis. According to Rico et al. (Rico et al. 2011), GLU is a primary excitatory neurotransmitter, whereas GABA is a key inhibitory neurotransmitter (McCormick 1989). According to Chandel et al. (Chandel et al. 2016), a fundamental concept in epileptogenesis is a disruption in the equilibrium between excitation and inhibition within a particular neuron or neuronal system, which results in excessive excitation and, ultimately, epileptic convulsions. PTZ, a potent GABA receptor antagonist, reduces GABA levels and GABA-A receptor density, leading to sustained cortical stimulation and seizure-like activity (Huang et al. 2001). Consequently, PTZ-induced convulsions may be eliminated by using substances that increase GABA levels, upsurge the density of GABA-A receptors, act as GABA-A receptor agonists (such as diazepam), or function similarly to GABA (Fisher et al. 2005). Numerous studies have reported altered neurotransmitter levels in the brains of PTZ-treated rodents, such as decreased GABA and increased GLU concentrations in the brain (Anwar et al. 2025; Koshal and Kumar 2016). Our findings confirmed decreased GABA and elevated GLU levels in PTZ-treated mice. In contrast, treatment with LLE and MβCD-NS (200 mg/kg) restored GABA levels and reduced GLU concentrations throughout the entire brain, suggesting a modulatory effect on GABAergic and glutamatergic neurotransmission that may underline their observed anticonvulsant activity.

In addition, epileptogenesis is defined as intricate anatomical alterations in the brain that transform a healthy brain into one that is impaired by frequent seizure activity (Reddy and Kuruba 2013). Similarly, massive neuronal degeneration has occurred in the hippocampal regions (CA and DG), and cerebral cortex of the PTZ-treated group, relative to the control mice, as shown by the histopathological examinations. Our results are in parallel to the outcomes of former research (Demyashkin et al. 2024; Nkwingwa et al. 2023), documenting the structural changes in brain regions in the case of epilepsy. Contrary to that, LLE or MβCD-NS (200 mg/kg bw) antagonized PTZ-induced neural damage in mice, highlighting its neuroprotective activity. These beneficial actions might be attributed to its active constituents.

Regarding the previous research concerning the correlation between neuroprotective activity and the major detected phytochemical classes: phenolic acids and flavonoids which represented 25 metabolites of the total identified compounds as presented in Table 1, it could be noted that flavonoids, in general, offer the most widely spread category of compounds with diversity of several subclasses such as flavones, flavonols, flavanols, etc., and their glycosides (Rio et al. 2013). Several identified compounds exhibit neuroprotective effects and can cross the blood–brain barrier (BBB). Notably, the lipophilic nature of apigenin facilitates its BBB penetration, contributing to its neuroprotective and anticonvulsant activities (Socała et al. 2025). Similarly, quercetin and kaempferol have shown neuroprotection in animal models of epilepsy primarily through antioxidant and anti-inflammatory mechanisms (Ahmed et al. 2021; Tavakoli et al. 2023). However, quercetin was documented to penetrate BBB (Youdim et al. 2004). Phenolic acids such as caffeic and chlorogenic also display neuroprotective potential by reducing oxidative stress and inflammation while enhancing antioxidant defenses (Althagafi 2024; Caruso et al. 2022; Coelho et al. 2015). Notably, chlorogenic acid’s BBB permeability has been demonstrated (Bai et al. 2023; Kumar et al. 2019). Hence, the corresponding antioxidant, anti-inflammatory, and neuroprotective activities of the extract could be strongly correlated to the synergistic effect of the identified compounds.

The superior effect of the nanosuspension under study can be due to the reduced particle size and enhanced oral absorption of the nanoparticles. Also, the inclusion of Kleptose Crysmeb^®^ as a nanosuspension-stabilizer can enhance the aqueous solubility of the active agents and can facilitate their penetration through biological membranes thereby improving their bioavailability. As the CD concentration increases, a greater amount of the active agent is solubilized, leading to an enhanced drug diffusion to the membrane surface (Rasheed 2008). Also, CD is a well-known penetration enhancer which can increase membrane permeability (Másson et al. 1999).

## Conclusion

The study conclusion implies that both LLE and MβCD-NS have significant protective properties against acute seizure, with superior effects of MβCD-NS at a dose equivalent to 200 mg extract/kg. MβCD-NS effectively dampened brain oxidative stress, and neuro-inflammatory response, as well as counteracted the disturbance of the brain neurotransmitters, which are key factors in its anticonvulsant action. In addition, MβCD-NS at the high dose level well-maintained the brain structure and function, as validated by the histopathological and behavioral assessments. The observed anticonvulsant effect of the extract might be attributed to a synergistic mechanism of its phytochemical constituents that possess significant neuroprotective properties.

### Study limitations and future plans

The present study employed an acute PTZ-induced seizure model, which primarily reflects short-term seizure protection rather than chronic epileptogenesis. Hence, the outcomes should be interpreted within the context of acute anticonvulsant activity. Further investigations using chronic or kindling seizure models are required to assess the long-term efficacy and antipileptogenic potential of LLE and MβCD-NS. Additionally, in-depth investigations are necessary to elucidate the specific molecular mechanisms underlying its neuroprotective activity. Future studies are planned to explore the specific molecular pathways involved, including GABAergic modulation, through targeted pharmacological and receptor-binding approaches. Moreover, detailed pharmacokinetic and tissue-distribution analyses could be a focus of future studies.

## Supplementary Information

Below is the link to the electronic supplementary material.

Supplementary Material 1 (DOCX 1.28 MB)

## Data Availability

Data is provided within the manuscript or supplementary files.
