# Supplementary material for: Metabolomics profiling and neuroprotective effects of Lagerstroemia loudonii leaf extract and its kleptose Crysmeb®- stabilized loaded nanosuspension in seizure mice model
Source: Metab Brain Dis. 2025 Dec 17;41(1):2. doi: 10.1007/s11011-025-01756-x (PMC12711940; doi:10.1007/s11011-025-01756-x)
Supplement: Supplementary file 1 — Supplementary Material 1 (DOCX 1.28 MB) [file 11011_2025_1756_MOESM1_ESM.docx]

**Metabolomics Profiling and Neuroprotective Effects of *Lagerstroemia loudonii* Leaf Extract and its Kleptose Crysmeb^®^- Stabilized Loaded Nanosuspension in Seizure Mice Model**

**
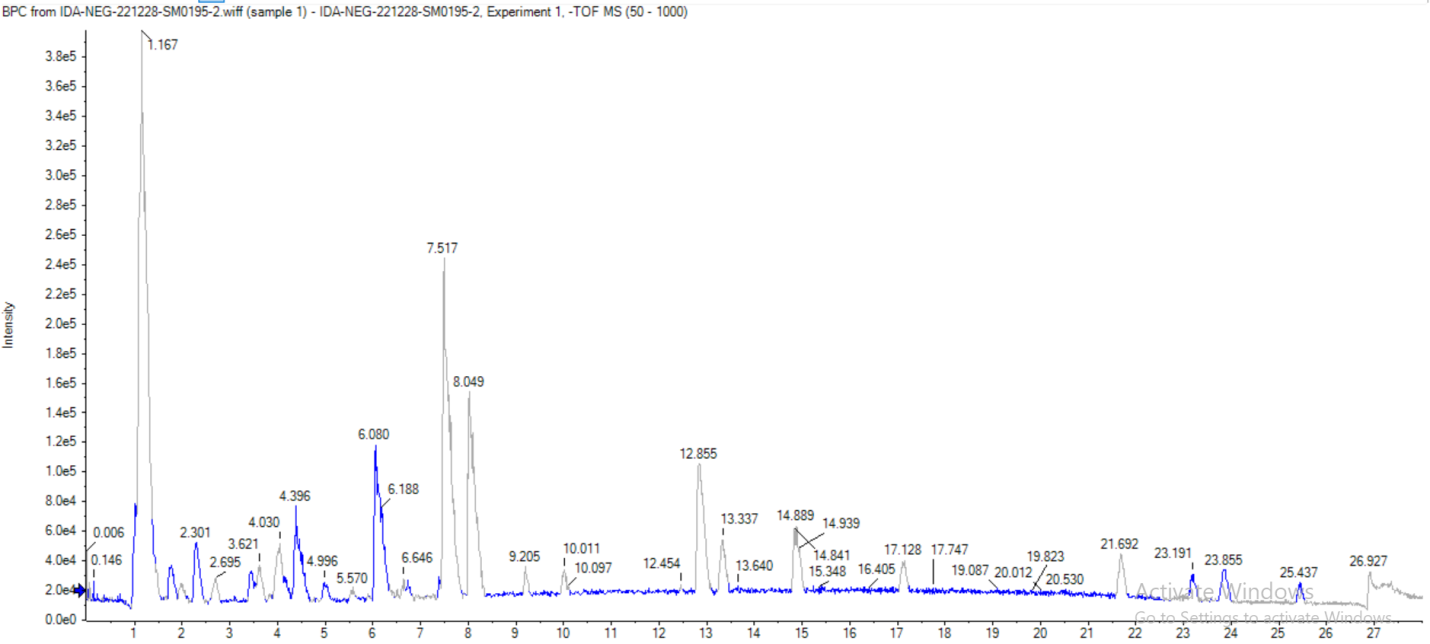
**

**Figure 1S: Base peak chromatogram in negative mode**

**
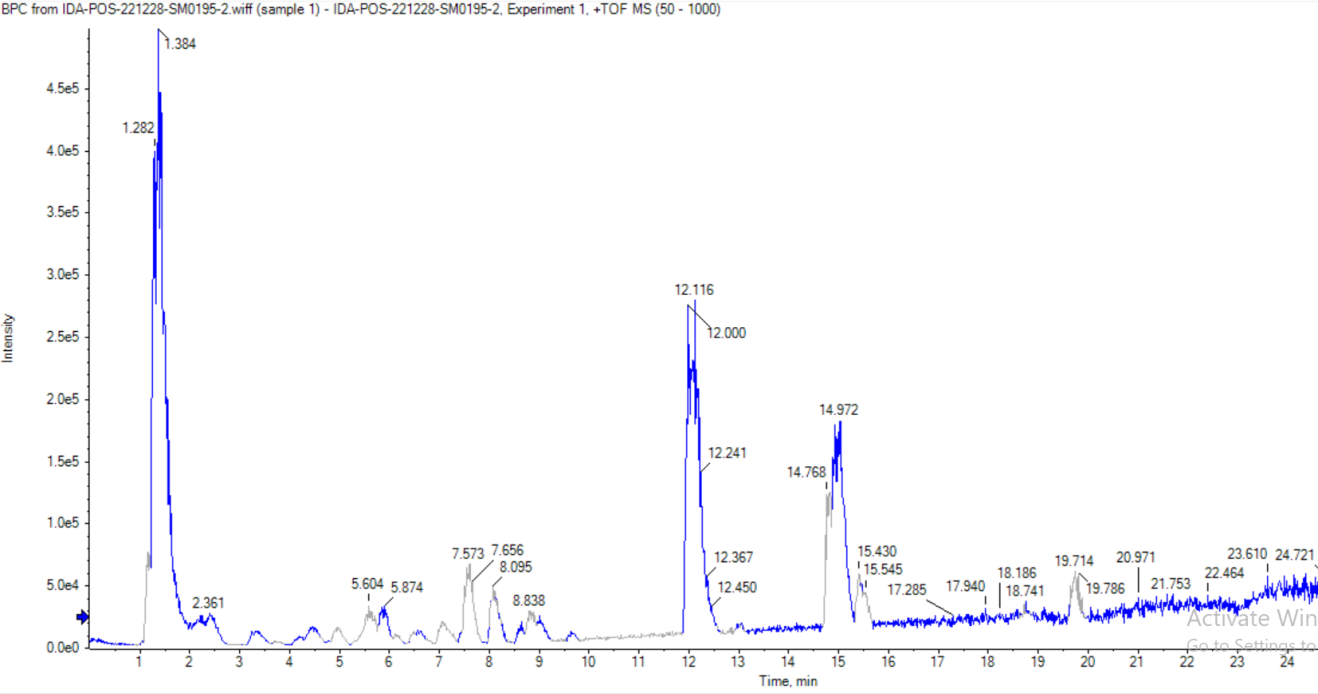
**

**Figure 2S: Base peak chromatogram in positive mode**

| 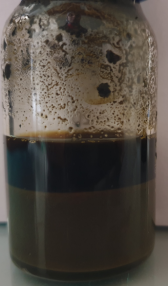  1% MβCD | 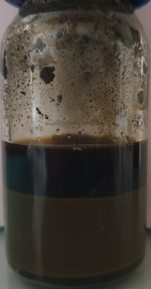  2% MβCD | 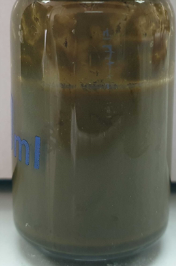  5% MβCD |
| --- | --- | --- |

**Figure 3S: Photos of the prepared MβCD-NS after storage.**

**
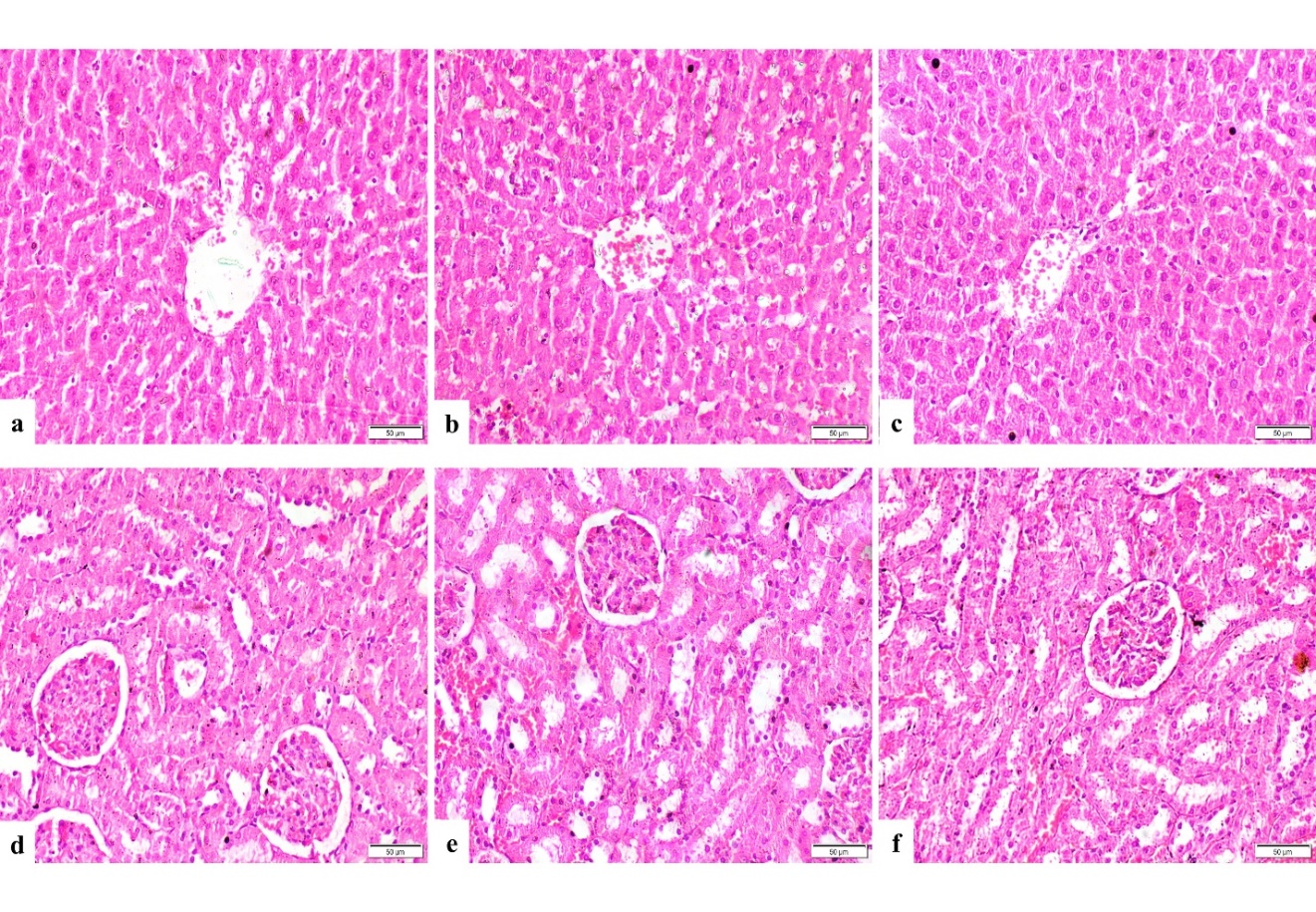
**

**Figure 4S (Supplementary file): Photomicrograph, rat liver and kidneys (scale bar 50 μm).** (a), (b) and (c) liver of normal, LLE, and MβCD-NS groups, respectively showing normal histological structure of hepatocytes. (d), (e) and (f) kidneys of normal, LLE, and MβCD-NS groups, respectively showing normal histological structure of renal tubules and renal corpuscles. LLE; ethanolic leaf extract of *Lagerstroemia loudonii*, MβCD-NS; extract-loaded MβCD-stabilized nanosuspension
